# Reduced genetic diversity of freshwater amphipods in rivers with increased levels of anthropogenic organic micropollutants

**DOI:** 10.1111/eva.13387

**Published:** 2022-05-10

**Authors:** Vid Švara, Stefan G. Michalski, Martin Krauss, Tobias Schulze, Stephan Geuchen, Werner Brack, Till Luckenbach

**Affiliations:** ^1^ Department of Effect‑Directed Analysis Helmholtz Centre for Environmental Research – UFZ Leipzig Germany; ^2^ 9173 Department of Evolutionary Ecology and Environmental Toxicology Goethe University Frankfurt Frankfurt am Main Germany; ^3^ 52732 UNESCO Chair for Sustainable Management of Conservation Areas Carinthia University of Applied Sciences Villach Austria; ^4^ Department of Community Ecology Helmholtz Centre for Environmental Research – UFZ Halle Germany; ^5^ Department of Bioanalytical Ecotoxicology Helmholtz Centre for Environmental Research – UFZ Leipzig Germany

**Keywords:** anthropogenic pollution, evolutionary toxicology, *Gammarus pulex*, LC‐HRMS, microsatellites, population genetics

## Abstract

Anthropogenic chemicals in freshwater environments contribute majorly to ecosystem degradation and biodiversity decline. In particular anthropogenic organic micropollutants (AOM), a diverse group of compounds, including pesticides, pharmaceuticals, and industrial chemicals, can significantly impact freshwater organisms. AOM were found to impact genetic diversity of freshwater species; however, to which degree AOM cause changes in population genetic structure and allelic richness of freshwater macroinvertebrates remains poorly understood. Here, the impact of AOM on genetic diversity of the common amphipod *Gammarus pulex* (Linnaeus, 1758) (clade E) was investigated on a regional scale. The site‐specific AOM levels and their toxic potentials were determined in water and *G*. *pulex* tissue sample extracts for 34 sites along six rivers in central Germany impacted by wastewater effluents and agricultural run‐off. Population genetic parameters were determined for *G*. *pulex* from the sampling sites by genotyping 16 microsatellite loci. Genetic differentiation among *G*. *pulex* from the studied rivers was found to be associated with geographic distance between sites and to differences in site‐specific concentrations of AOM. The genetic diversity parameters of *G*. *pulex* were found to be related to the site‐specific AOM levels. Allelic richness was significantly negatively correlated with levels of AOM in *G*. *pulex* tissue (*p* < 0.003) and was reduced by up to 22% at sites with increased levels of AOM, despite a positive relationship of allelic richness and the presence of waste‐water effluent. In addition, the inbreeding coefficient of *G*. *pulex* from sites with toxic AOM levels was up to 2.5 times higher than that of *G*. *pulex* from more pristine sites. These results indicate that AOM levels commonly found in European rivers significantly contribute to changes in the genetic diversity of an ecologically relevant indicator species.

## 1 INTRODUCTION

Chemical pollution, river regulation, and the invasion of alien species degrade freshwater ecosystems worldwide (Grizzetti et al., 2017; Ormerod et al., 2010; Rohr et al., 2006; Vörösmarty et al., 2010). This degradation becomes evident by biodiversity declines on both local and global scales (Thieme et al., 2010), as a third of all freshwater species faces a high extinction risk (Collen et al., 2014). In particular, anthropogenic organic micropollutants (AOM) were recognized as one of the major drivers of the biodiversity declines in freshwaters (Liess & von der Ohe, 2005; Malaj et al., 2014; Münze et al., 2017). AOM include bioactive compounds, such as pesticides (Pimentel, 2009) and pharmaceuticals (Daughton & Ternes, 1999; Ginebreda et al., 2010) that mostly pass standard water treatment in wastewater treatment plants (WWTP) and are discharged into rivers and streams where they accumulate in the habitats of freshwater organisms and in their tissues (Stamm et al., 2016). AOM in the environment were shown to impact species distribution, freshwater community composition, and the species’ ecological function (Burdon et al., 2019; Englert et al., 2017; Liess & von der Ohe, 2005; Peschke et al., 2014). The effects of AOM across different levels of the biological organization do not only affect species distribution and function but also drive the evolution of species by affecting intraspecific genetic diversity and traits in exposed populations (Bickham et al., 2000).

The effects of AOM on genetic diversity in exposed natural populations are diverse and often difficult to predict due to the immense diversity of AOM that enter freshwater ecosystems (Brown et al., 2009). Different AOM can modify the genetic diversity of species in direct and indirect ways. AOM directly affecting a species’ gene pool comprise genotoxic and mutagenic chemicals with the potential to modify DNA integrity (Bickham, 2011). Such AOM, for instance antineoplastic agents and aromatic amines (Muz et al., 2017; Steger‐Hartmann et al., 1997), can alter DNA replication and chromosome structure and can cause nucleotide substitutions, deletions, or duplications (Devaux et al., 2011; Lacaze et al., 2011; Theodorakis et al., 2001). The emergence of new genetic variants may increase the genetic diversity of species, but it can also lead to deleterious mutations causing reduced reproductive fitness (Bickham et al., 2000). In addition to direct genetic effects, AOM can cause changes in the genetic diversity of a species in an indirect way by affecting species fitness. In exposed species, selective effects of AOM may promote specific genotypes by adverse short‐term or sublethal long‐term effects (Brown et al., 2009). Short‐term effects, for example by pesticides, can result in high mortality rates that increase genetic drift (Coors et al., 2009; Coutellec et al., 2013) or select genotypes resistant to a direct specific toxic impact (Bell & Gonzalez, 2009). Long‐term exposure to sublethal AOM levels can not only cause effects such as reduced mobility or feeding ability (Englert et al., 2017; Nyman et al., 2013), but also promote species traits associated with increased resistance to AOM.

Genetic shifts in species impacted by AOM can be associated with altered genetic diversity parameters, such as reduced allelic richness or an increased inbreeding rate (Brown et al., 2009). In populations adapted to exposure to toxic AOM, the overall allelic richness can be reduced with increased frequency of certain alleles associated with resistance to pesticides with specific modes of action (Bickham et al., 2000). For example, tolerance‐related alleles were found to be prevalent in *Hyallela azteca* (Saussure, 1858) living in habitats contaminated with pyrethroid insecticides and rare in *H*. *azteca* from nonpolluted sites (Weston et al., 2013). In addition to adaptation‐related alleles, genetic change of species living in environments with toxic contaminants is associated with reduced genetic diversity rates in genetic markers that are not necessarily related to specific adaptive change (Brown et al., 2009). For instance, the diversity of alleles was reduced in marine and terrestrial amphipods originating from sites with sediments polluted with polyaromatic hydrocarbons (PAHs) and heavy metals (Bach & Dahllöf, 2012; Ungherese et al., 2010).

Increased levels of AOM are not exclusively associated with the reduction of genetic diversity in natural environments. In very mobile species, changes in genetic diversity may be compensated by gene flow from sites without AOM (Lenormand, 2002). Furthermore, mutagenic AOM can significantly increase the genetic diversity of exposed populations (Eeva et al., 2006). For example, increased genetic diversity in redbreast sunfish, *Lepomis auritus* (Linnaeus, 1758), was associated with the presence of mutagenic chemicals from toxic paper mill effluents (Theodorakis et al., 2006). A species’ genetic diversity can also be enhanced by the presence of AOM with different modes of action (Whitehead et al., 2017). Thus, in contrast to, for example pesticides directly impairing physiological fitness of exposed species, endocrine disruptors can cause shifts in genetic inheritance, for example by altered gametogenesis (Alves da Silva et al., 2018; Coulaud et al., 2015; Xuereb et al., 2011).

Despite multiple evidences for changes in the genetic diversity of species exposed to environmental pollutants, such as mutagenic chemicals, heavy metals, or PAHs (Bach & Dahllöf, 2012; Theodorakis et al., 2006; Ungherese et al., 2010; Weigand et al., 2018), data on genetic changes in freshwater macroinvertebrates exposed to AOM in rivers are scarce. In most cases, studies on AOM’s impact on the genetic diversity of freshwater macroinvertebrates investigated alterations at the local scale (Inostroza, Vera‐Escalona, et al., 2016; Inostroza, Wicht, et al., 2016; Švara et al., 2021). In a recent study investigating the frequencies of specific alleles to reveal the genetic structure of *Gammarus pulex* (Linnaeus, 1758) sampled along a pollution gradient in a river, the physiological condition of the amphipods was found to depend on AOM contamination at the respective sampling site, while the genetic structure of *G*. *pulex* within the river did not show AOM‐dependent changes (Švara et al., 2021). However, due to the limitations of the study performed in a single river with a few sampling sites, the relationship between the genetic diversity of *G*. *pulex* and different levels of AOM in rivers remains unclear.

To expand upon the previous results and comprehensively determine the association between the genetic diversity of species and different levels of AOM, genetic diversity parameters of the amphipod species *G*. *pulex* and site‐specific AOM profiles were here investigated on a regional scale in central Germany. We compiled and examined a comprehensive data set comprising data on AOM concentrations in water and *G*. *pulex* tissue, AOM toxicity levels for *G*. *pulex*, and several *G*. *pulex* genetic diversity parameters for 34 sites across six rivers from three catchment areas. The study sites were selected based on criteria assumed to result in the diversity of study sites with regard to pollutant levels. We selected different locations along a stream course and considered the absence/presence of effluents from wastewater treatment plants (WWTP) and run‐offs from agricultural and urban areas. In the previous study on the genetic divergence of *G*. *pulex* from polluted and nonpolluted sites within a river, *G*. *pulex* individuals from the different sites were found to not be genetically different (Švara et al., 2021). Thus, we hypothesize that (1) divergence in population genetic structure within rivers and across rivers in a region corresponds to distances between sites rather than to different levels of toxic AOM in the rivers. In contrast to divergence in species genetic structure, increased levels of toxic AOM at the sites downstream of the main sources of pollution (e.g., WWTP, agriculture) likely exert selective pressure on amphipods and affect species genetic diversity parameters. Therefore, we expect (2) AOM‐related reduction of allelic richness and effective population size along with an increase in the inbreeding rates of *G*. *pulex* at sites with high AOM concentrations and toxicity levels in the studied region.

## 2 MATERIALS AND METHODS

### 2.1 Study sites and sampling

**FIGURE 1:**
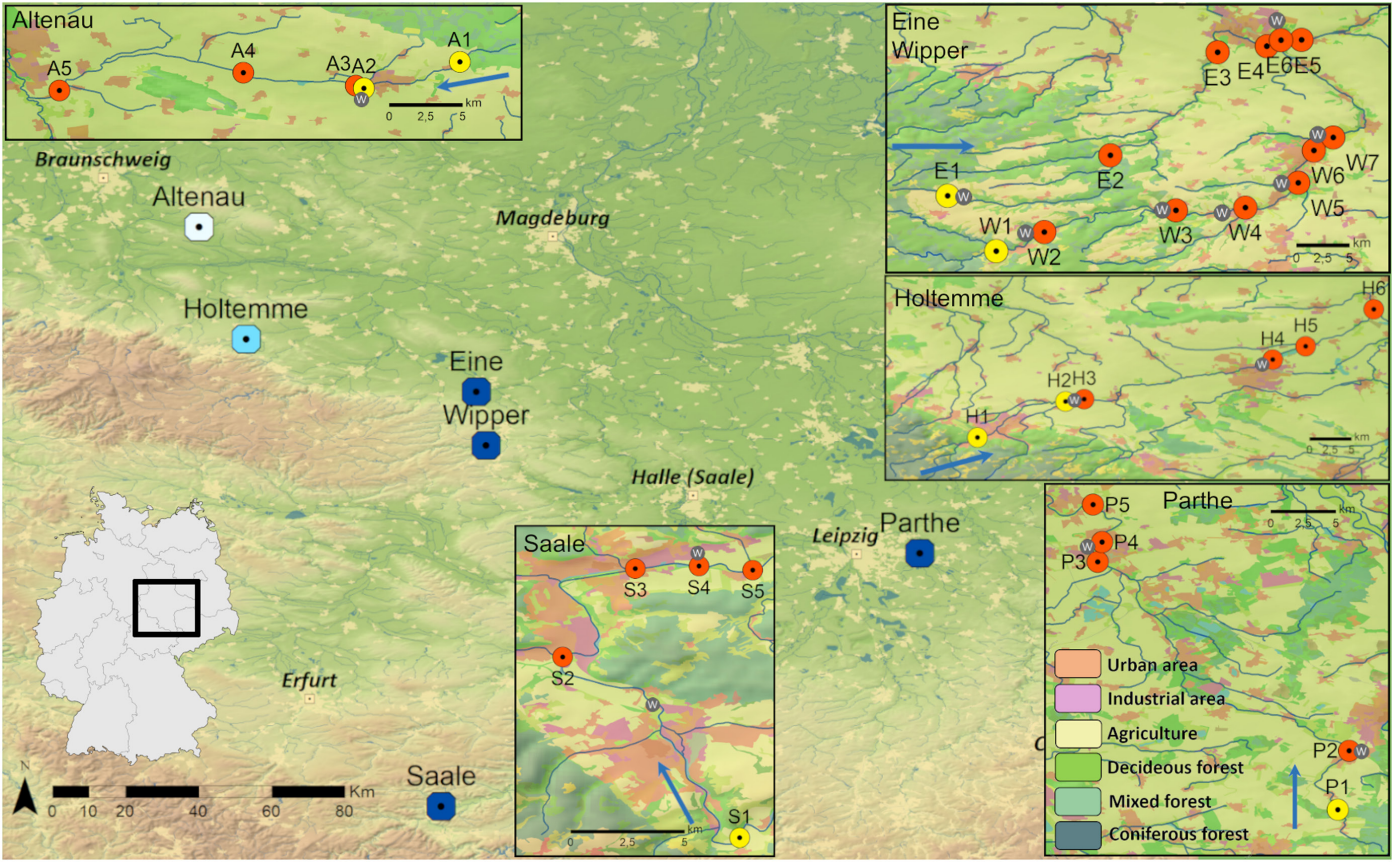
Map of the studied region with the six rivers and the sampling sites. The region is indicated by a square on the map of Germany (bottom left). The large map shows an overview of the region with the studied rivers and the major cities marked for orientation. Symbols colored in different shades of blue indicate different catchments: Oker River catchment—white, Bode River catchment—light blue, and Saale River catchment—dark blue. The studied sites are shown in the panels with each sampling site marked with colored circles (A—the Altenau River, E—the Eine River, H—the Holtemme River, P—the Parthe River, S—the Saale River, and W—the Wipper River). The colors of the circles in the detailed maps indicate whether a wastewater treatment plant (WWTP) is located upstream of the study site (yellow—no WWTP; red—WWTP). Grey circles with a white “W” indicate the locations of WWTP. Blue arrows indicate the direction of the waterflow. Land use in the areas of the detailed maps is indicated by the colors explained in the legend in the bottomright corner of the figure: green colors—different forest types, red—urban areas, purple—industrial areas, and yellow—agricultural areas. The length of the scale bars in the panels indicates 5 km

Samples for genetic and chemical analyses were taken at eight reference sites (upstream of settlements and WWTP) and 26 AOM‐polluted sites along six rivers (Altenau River (A), Eine River (E), Holtemme River (H), Parthe River (P), Saale River (S), Wipper River (W)) belonging to three catchments in the states of Lower Saxony, Saxony, Saxony‐Anhalt, and Thuringia in central Germany (Figure 1). The rivers flow through forest, urban, and agricultural landscapes with run‐offs and WWTP effluents as the main sources of anthropogenic water contaminants. The numbers of WWTP located at the analyzed river stretches were: one at the Altenau River, two at the Eine River, two at the Holtemme River, two at the Parthe River, two at the Saale River, and five at the Wipper River (Figure 1). At each site, the common Palearctic amphipod species *G*. *pulex* was collected along with water samples. The chemical analysis of the two types of samples enabled detection of a broad spectrum of freely dissolved and tissue‐bound, potentially ecotoxicologically relevant AOM. In parallel to the sample collection, water parameters comprising temperature, pH, O_2_ concentration, and conductivity were measured (Table S1). *Gammarus pulex* amphipods were caught by kick‐net sampling (0.5 mm mesh size) across the whole width of a river with at least five locations per sampling site. Sampled amphipods were morphologically identified using a taxonomical key (Altermatt et al., 2019). The abundance of *G*. *pulex* at each site was estimated by the recorded number of individuals per catch. Amphipods for chemical analysis were rinsed with distilled water at the sampling site, dried on a clean paper towel, and stored at −20°C until analysis. For DNA analysis, *G*. *pulex* specimens were stored in absolute ethanol. One milliliter of river water samples were collected and frozen at −20°C until chemical analysis. For detailed information on the sampling sites, refer to Table S1.

### 2.2 AOM quantification and toxicity estimation

AOM concentrations in water and *G*. *pulex* tissue samples were analyzed by a Thermo Ultimate 3000 liquid chromatography (LC) system coupled with a quadrupole‐orbitrap high‐resolution mass spectrometer (HRMS; Thermo QExactive Plus) as described in Švara et al. (2021). For details on sample preparation and LC‐HRMS measurement refer to Section S1 in the Supporting Information.

To quantify AOM in the samples, raw data from the LC‐HRMS analysis were converted into the.mzML format using ‘ProteoWizard v3.0.18265’. The peak list for each batch was generated by MZmine v2.32 (Pluskal et al., 2010) with settings as suggested by Beckers et al. (2020). The list was annotated for 523 compounds from water samples and 497 compounds from *G*. *pulex* tissue samples and corrected for blanks. The analyzed compounds comprised AOM with a wide spectrum of hydrophobicity and application categories including pesticides, pharmaceuticals, and household and industrial chemicals known to occur in Central European rivers regularly (Beckers et al., 2018; Inostroza, Vera‐Escalona, et al., 2016; Inostroza, Wicht, et al., 2016; Munz et al., 2017). Refer to S1.4 in the Supporting Information for details on data evaluation and to Table S2 for a list of analyzed compounds.

The toxic potential of the analyzed AOM was estimated by converting the measured *G*. *pulex* tissue concentrations into toxic units (TU). TUs were calculated based on the lethal concentrations for 50% of individuals in standard toxicity tests (LC_50_) for the respective compounds given in the EPA ecotoxicology database (https://www.epa.gov/chemical‐research/ecotoxicology‐database). Data from the database were retrieved as a text file (exotox_ascii_15_09_2020) and the mean was calculated from all LC_50_ values obtained in 24 or 48 h exposure experiments for *G*. *pulex* for a respective compound. If no LC_50_ data for a compound were available for *G*. *pulex*, LC_50_ data for *Daphnia magna* Straus, 1820 were used. The freely dissolved fraction (*C* ^fd^) of each compound *i* was estimated based on the measured tissue concentrations according to the equilibrium partitioning theory using the following equation:

$$C_{i}^{\text{fd}}=\frac{C_{i}^{\text{tG}}}{f_{\text{LIPID}}D_{\text{OW}}}$$

where *C* ^tG^ is the total measured concentration [ng/g of wet tissue] of a compound in *G*. *pulex*; *f*_LIPID_, the lipid fraction value predicted for *G*. *pulex* by Ashauer et al. (2010; 1.34% of total body weight); and *D*_ow_, the n‐octanol‐water distribution coefficient. The coefficient values were calculated using ACD Perfecta 2014. The potential toxicity of the individual AOM for *G*. *pulex* was determined by calculating TU values (Table S5). The TUs for all compounds in an extract were summed to predict the potential for an additive adverse effect of those chemicals, as described in Švara et al. (2021):

$$\log\sum \text{TU}=\log\sum \left(\frac{C_{i}^{\text{fd}}}{{\text{LC}}_{50,i}}\right)$$

### 2.3 Assessment of *Gammarus pulex* genetic diversity and structure

To assess the population genetic structure and genetic diversity parameters of *G*. *pulex*, 16 microsatellite loci were genotyped in a total of 931 individuals from the different sampling sites (10–30 individuals per site; Table S8). Genomic DNA was extracted from pereopods of each individual using the DNeasy Blood & Tissue kit (Qiagen). DNA integrity was checked on an agarose gel, followed by DNA concentration quantification using a NanoDrop spectrophotometer (NanoDrop Technologies Inc.). A segment of the mitochondrial cytochrome oxidase I (COI) gene from randomly selected individuals (>5 per river) was sequenced to assess whether *Gammarus* cryptic lineages were present in the studied region. For details on polymerase chain reaction parameters, sequencing conditions, and primers refer to Švara et al. (2021) and Table S6.

Microsatellite loci (Table S7) were amplified and genotyped from 20 (Holtemme River sites) to up to 30 (all other sites) *G*. *pulex* DNA samples following the protocol described in Švara et al. (2019). Microsatellite genotype data for each individual and each locus with missing genotype information (>20%) were removed from the data set. Null alleles and deviations from Hardy‐Weinberg equilibrium were assessed using the R package ‘popgenereport’ (Adamack & Gruber, 2014). Subsequently, rarefied allelic richness and private allele rates were calculated in ‘HP‐Rare’ (Kalinowski, 2005) for *G*. *pulex* from each sampling site, followed by the estimation of observed (H_o_) and expected (H_e_) heterozygosity and the inbreeding coefficient (F_IS_) using the R package ‘Hierfstat’ (Goudet, 2005). Pairwise differentiation and its statistical significance among *G*. *pulex* from different sampling sites and rivers were estimated by fixation index (*F*_st_) values, with the deviation from zero tested by applying 10,000 permutations of the analyzed loci. The effective population size at each site was estimated with the linkage disequilibrium model in ‘NeEstimator 2.0.2’ (Do et al., 2014) and an alpha value of less than 0.05. Hierarchical variance significance of the genetic differentiation among sites and rivers was calculated by analysis of molecular variance (AMOVA) in the R package ‘Poppr’ (Kamvar et al., 2014).

To assess the composition of genotypes in the rivers, a population structure analysis was performed in Structure 2.3.4. (Raj et al., 2014). An admixture model without any a priori information was run 10 times for clusters K from 1 to 10 using 1,200,000 MCMC steps and discarding the first 200,000 steps as a burn‐in. The optimal number of clusters was determined in Structure Harvester (Earl & von Holdt, 2012) with the Evanno method (Evanno et al., 2005). Using CLUMPP 1.1.2. (Jakobsson & Rosenberg, 2007), the runs were merged into a single plot and visualized in DISTRUCT 1.1. (Rosenberg, 2004).

The association between distance and genetic differentiation among sites was tested using the Mantel test by comparing pairwise *F*_st_ values and waterway distances between all sites for regional comparison and sites within each river for local comparison. Distances between sites were estimated using the ‘network analyst toolbox’ in ArcGIS (ESRI). The impact of environmental pollution on genetic differentiation between sites was assessed by partial Mantel tests correlating pairwise *F*_st_ values against distances based either on total AOM levels or TUs, while accounting for the effect of waterway distances.

### 2.4 Analyses of AOM relation to genetic diversity parameters

Relations between AOM levels and amphipod genetic diversity indicators, including allelic richness, private allele rates, inbreeding coefficients (*F*_IS_), effective population size (N_e_), and *G*. *pulex* abundance, were analyzed by linear mixed‐effect models (LME). Genetic diversity indicators were explained by linear fixed effects of distance from the spring, toxic units based on the AOM from *G*. *pulex* tissue samples, total concentration of detected AOM in amphipod tissue, presence of WWTPs before the sampled sites, conductivity, pH, and saturation of water with oxygen, allowing a random intercept for each river. The values of the total concentration of detected compounds and effective population sizes were log‐transformed to avoid the effects of very low values close to 0. The sites, for which, due to limited numbers of *G*. *pulex*, AOM‐tissue concentrations were not available, were excluded from the analysis (i.e., S2, W5, W6). Using the function lmer in the package ‘lme4’ (Bates et al., 2015), a global model including all environmental site characteristics was constructed. To select for the fixed effects contributing to differences in the analyzed indicators, the best fitting models were selected based on the lowest Akaike information criterion (AICc; delta AICc of less than 5 were considered) and the highest log‐likelihood using the dredge function from the package ‘MuMIn’ in R (Burnham & Anderson, 2002).

Structural equation models (SEM) were used to fit allelic richness against total AOM concentration and distance from the source for all sites in each river. The models were fitted by generalized least squares by applying the sem function from the R package ‘lavaan’ (Rosseel, 2012). In the global model for all rivers, TUs and *G*. *pulex* abundance were included as intermediate explanatory variables, as differences in total concentrations of AOM could be reflected in TU values and in *G*. *pulex* abundances.

## 3 RESULTS

### 3.1 AOM detected in water and *Gammarus pulex* tissue samples

**FIGURE 2:**
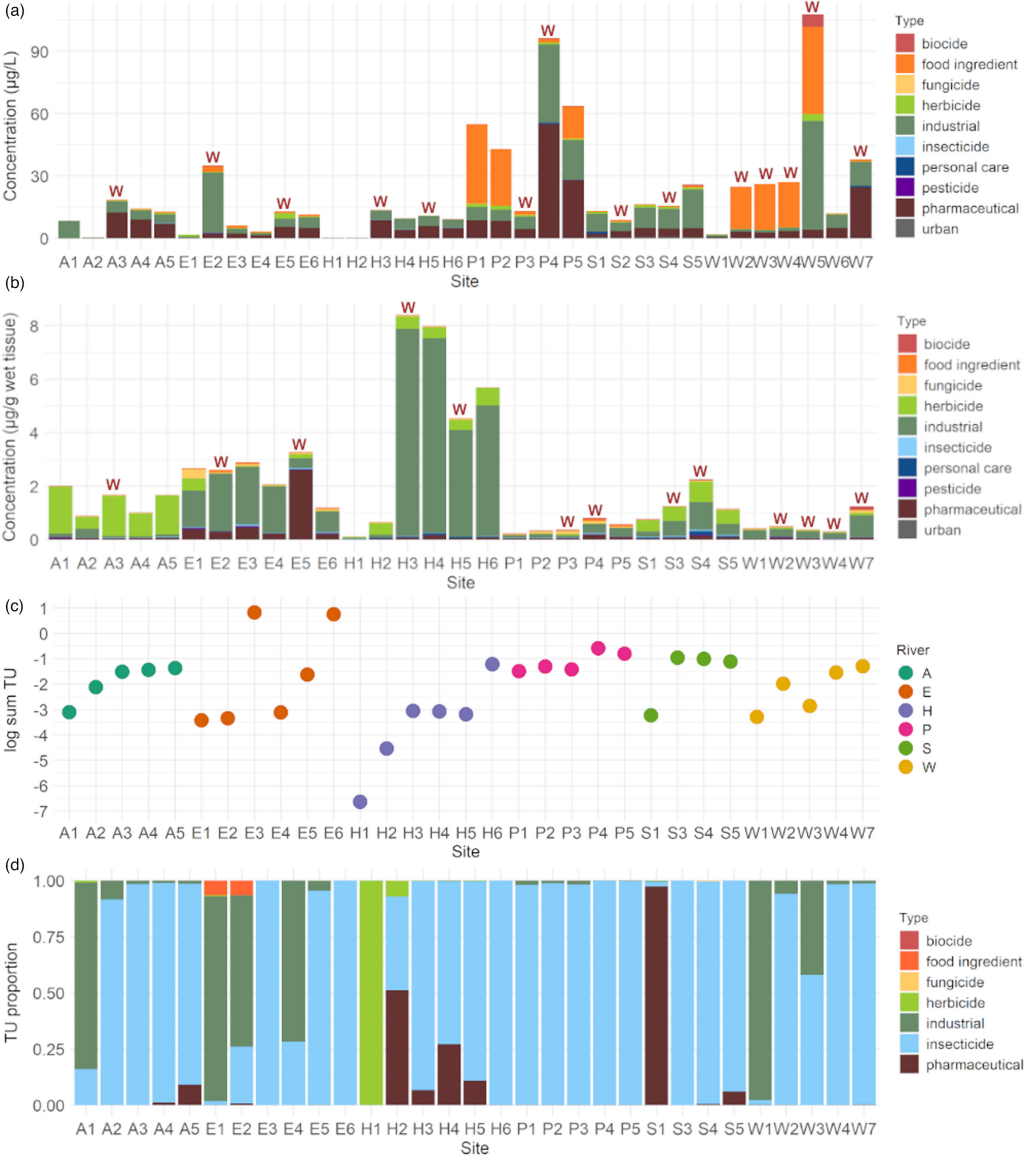
Levels of AOM and their toxicities for *Gammarus pulex*. (a) Total concentrations of AOM from different application types (types are marked by different colors) in water samples from each site. (b) Total concentrations of different AOM types measured in *G*. *pulex tissue* samples from each site. (c) Toxic units (TU) calculated for AOM found in *G*. *pulex* tissue samples from each site. (d) TU ratio of AOM with different application types based on AOM concentrations measured in *G*. *pulex* tissue levels from each site. The sites situated directly downstream of WWTP effluents are marked with a red “W”

Numbers of detected AOM and their concentrations in the water samples indicated different site‐specific pollution patterns. In total, 236 compounds were detected in water samples from 34 sites. Most compounds were found in water samples from sites P4, S5, P5, and W7 with 152, 131, 130, and 104 compounds, respectively. The highest total AOM concentrations were 107.8 and 96.4 µg/L in water from sites W5 (Wipper River) and P4 (Parthe River), respectively (Figure 2a). Total AOM concentrations were lowest in water from sites H1 and H2 (0.2 and 0.4 µg/L, respectively; Holtemme River) and A2 (0.5 µg/L; Altenau River). From the analyzed AOM, the industrial chemical 1H‐benzotriazole (47.2 µg/L at site W5), the pharmaceutical theophylline (41.1 µg/L at site W5 and 37.4 µg/L at site P1), and the metformin transformation product guanylurea (14.8 µg/L at site P4) showed the highest concentrations. The herbicide metazachlor, the sweetener acesulfame (both detected at all 34 sites), the industrial chemical melamine, and the sweetener cyclamate (both detected at 33 sites) were found at most sites. Several compounds were found in water samples from only a single site (refer to Table S3 for an overview). Among the detected AOM, suspected carcinogens tris(2‐chloroethyl)phosphate at sites S5, E2, E5, and P4 and melamine at 31 sites were detected in the studied rivers (Table S3).

In *G*. *pulex* tissue samples, a total of 253 compounds were found at 31 sites. Most compounds were detected in tissue samples from sites E6 (155 compounds), P4 and P5 (109 compounds), E5 (103 compounds), and E3 and E4 (83 compounds). The highest (8.4 µg/g at H3) and the lowest (0.1 µg/g at H1) total AOM concentrations were detected in tissue samples from the Holtemme River (Figure 2b). The biocides benzyldimethyldodecylammonium and didecyldimethylammonium, the herbicide pendimethalin, and the surfactant decylsulfate were detected in tissue samples from all 32 sites. 7‐(ethylamino)‐4‐methylcoumarin (up to 7.6 µg/g at H3), tetrapropylammonium (up to 1.8 µg/g at E3), and pendimethalin (up to 1.5 µg/g at A1) were found at the highest concentrations among compounds detected in tissue extracts. The lowest number of compounds was detected in *G*. *pulex* tissue from sites H1, A1, and A2 (19, 43, and 49, respectively). Several compounds were detected in tissue samples from only one site; for detailed information see Table S4. The total AOM concentration generally increased at sites downstream of a WWTP effluent entering the river, yet, it did not linearly increase with distance from the first site (e.g., in the Altenau or the Eine Rivers) and at the sites downstream of the WWTP effluents (e.g., in the Altenau River). Among the detected AOM, a pharmaceutical with a genotoxic potential, tamoxifen, and its metabolite 4‐hydroxytamoxifen were found at sites S2 and S4 and at sites P4, W2, and W3 (Saale, Parthe, and Wipper Rivers). The potential mutagen carboline was detected at sites E2, E6, and P1 (Eine and Parthe Rivers). Suspected carcinogens tris(1,3‐dichlorisopropyl)phosphate (TDCPP; at sites E4, S4) and tris(2‐chloroethyl)phosphate (at sites H2–H5, E1–E4, E6, P1–P4) were measured in *G*. *pulex* tissue extracts (Table S4).

The differences among concentrations of AOM in water samples from the same river were larger than those measured in *G*. *pulex* tissue samples from the same river (Figure 2a,b). While AOM concentrations in water samples were highest for pharmaceuticals, food ingredients, industrial chemicals, and biocides, AOM concentrations in *G*. *pulex* tissue were highest for industrial chemicals, herbicides, pharmaceuticals, and fungicides. The correlation of the AOM concentrations in water and *G*. *pulex* tissue samples was rather low, with a Pearson's correlation coefficient of 0.28 across all sites.

### 3.2 Site‐specific toxic potentials of AOM

The TU values calculated from *G*. *pulex* tissue concentrations indicated an increased toxic potential downstream of the major pollution sources in the studied rivers. The sites with the highest TU values were either the ones located the furthest downstream at the sampled river stretches or the ones located downstream of the WWTP effluents and include sites from the Eine River (E3 at 6.74, E6 at 5.75), the Parthe River (P4 at 0.26, P5 at 0.16), and the Saale River (S3 at 0.11, S4 at 0.10, S5 at 0.08; Figure 2c). The lowest potentials for adverse effects from AOM in amphipods (TU <0.001) were indicated at sites upstream of WWTP effluents and run‐offs from agricultural areas (H1, H2, E1, E2, W1, and A1).

TU values were assessed for 44 AOM (Table S5) and were highest for the organophosphate transformation product 3,5,6‐trichloro‐2‐pyridinol, insecticides acetamiprid and imidacloprid, and the pharmaceutical acetaminophen. The insecticide 3,5,6‐trichloro‐2‐pyridinol was detected in two samples (E3, E6); respective TUs were > 0. The neonicotinoid insecticide acetamiprid was detected at 17 sites. At sites P4, P5, S3, and S4, the TU attributed to acetamiprid exceeded 0.01, the value known to cause acute effects in crustaceans (Malaj et al., 2014). Several other AOM, including insecticides (e.g., imidacloprid) and a pharmaceutical (i.e., acetaminophen), exceeded 0.001 TU (Table S5), and thus the threshold for chronic adverse effects for *G*. *pulex* (Malaj et al., 2014).

Acute and chronic TU levels were mostly attributed to insecticides (acetamiprid, imidacloprid, thiacloprid, clothianidin) and their transformation products, often contributing more than 95% of the total TUs (Figure 2d, Table S5). Another compound group that contributed significantly to the toxicity at the polluted sites included pharmaceuticals (e.g., acetaminophen and citalopram). Some AOM groups did not exceed the threshold for chronic toxicity, yet they significantly contributed to total TU. For example, at the sites with low TUs, industrial chemicals (A1, E1, E2, E4, W1, and W3), herbicides (H1 and H2), pharmaceuticals (H2, H4, and S1), and even food ingredients (E1 and E2) significantly contributed to the total TUs.

### 3.3 Genetic diversity and structure of *Gammarus pulex*

Based on the analyzed COI segment sequences, *G*. *pulex* samples from the studied rivers belonged to a single genetic lineage. Similarities of all sequences were greatest with those from *G*. *pulex* samples from the Brandenburg region (Figure S1). Therefore, all sampled *G*. *pulex* individuals could be assigned to clade E (Figure S1), according to the classification of Grabner et al. (2015).

In total, 931 *G*. *pulex* individuals were genotyped using microsatellites; upon quality control, data from 928 individuals were further analyzed. Across all 16 analyzed microsatellites, 138 different alleles were detected. All loci were polymorphic in *G*. *pulex* from the six rivers (Table S8). Averaged rarefied allelic richness per locus was highest in the Parthe River (2.98) and lowest in the Holtemme River (2.69; Table S8); ranges were 2.74 to 2.89 in the Altenau River, 2.34 to 2.89 in the Eine River, 2.43 to 2.88 in the Holtemme River, 2.75 to 2.99 in the Parthe River, 2.76 to 2.94 in the Saale River, and 2.61 to 2.85 in the Wipper River. The numbers of river‐specific private alleles per locus were highest in the Saale River (0.32) and lowest in the Wipper and the Parthe Rivers (0.18). No significant linkage between loci was detected when considering all sites. Null alleles were consistently detected for locus gp37, which was excluded from the structure analysis (Table S9). Observed heterozygosity across sites varied from 0.23 at the site directly at the most downstream WWTP at the Wipper River (W6) to 0.40 at the furthest upstream site at the Saale River (S1; Table S8). Expected heterozygosity varied from 0.35 at W6 to 0.44 at the site downstream of the WWTP at the Saale River (S5) and at the Parthe River (P3). The F_is_ values were lowest at upstream sites of rivers, including sites H1 and H2 at the Holtemme River and site S1 at the Saale River with −0.017, 0.026, and 0.073, respectively. The F_is_ values were highest mostly at more downstream sites. For example, F_is_ = 0.439 and F_is_ = 0.282 at sites W4 and E6 in the Wipper and the Eine Rivers, respectively (Figure S3).

**FIGURE 3:**
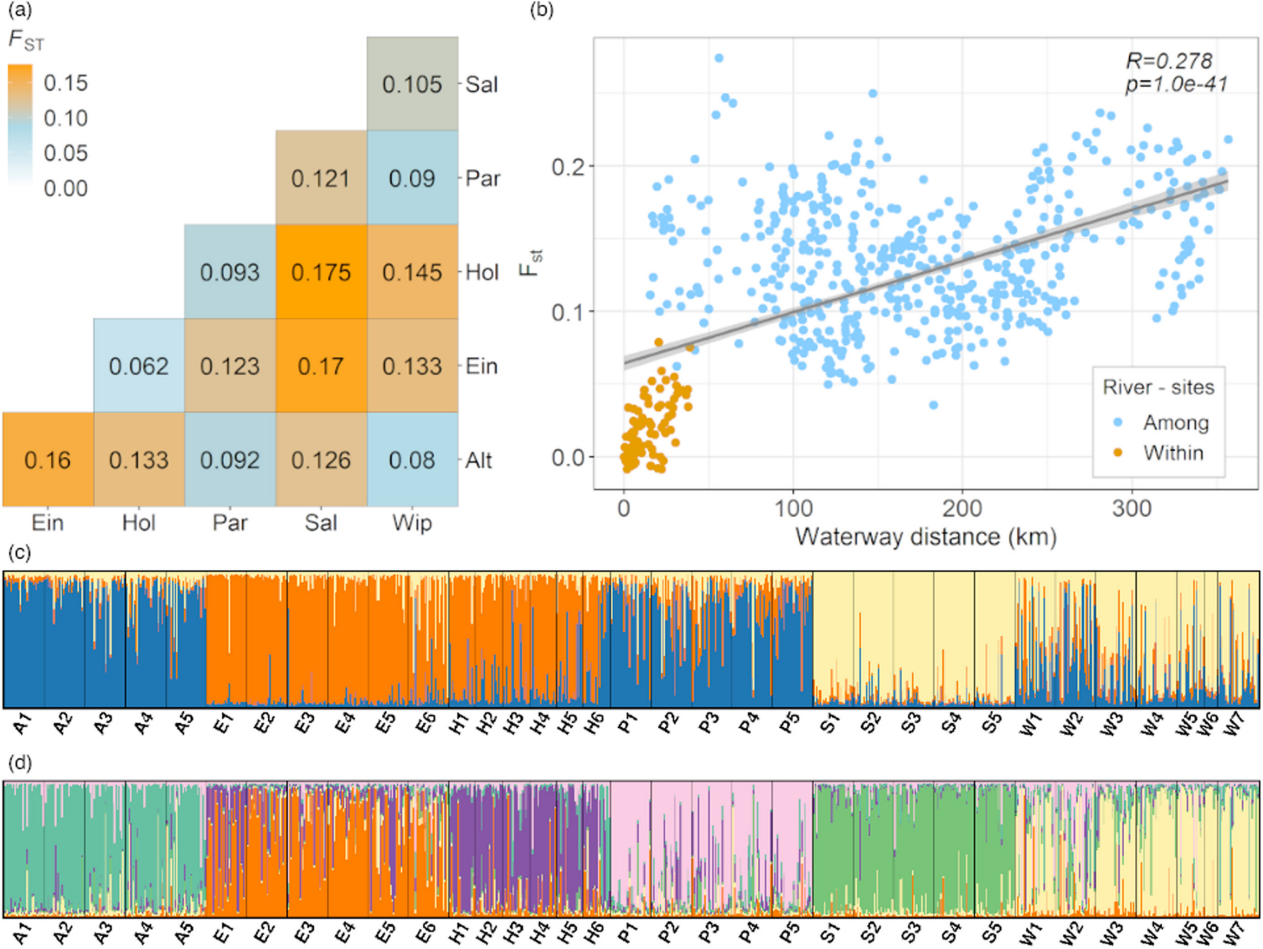
Genetic differentiation of *Gammarus pulex* in the studied rivers. (a) Pairwise *F*_st_ values estimated based on pooled *G*. *pulex* genotypes from different sites belonging to each river. All values are significantly different from zero. (b) Mantel test of pairwise *F*_st_ values between every pair of sites and respective waterway distances. Orange dots indicate pairwise comparison of sites within each river. Blue dots indicate pairwise comparison of sites among rivers. Structure analysis of *G*. *pulex* from 34 sites in six rivers with memberships/ancestry proportion to different clusters (c) K = 3 and (d) K = 6. Each vertical line represents a genotype of a single amphipod

**FIGURE 4:**
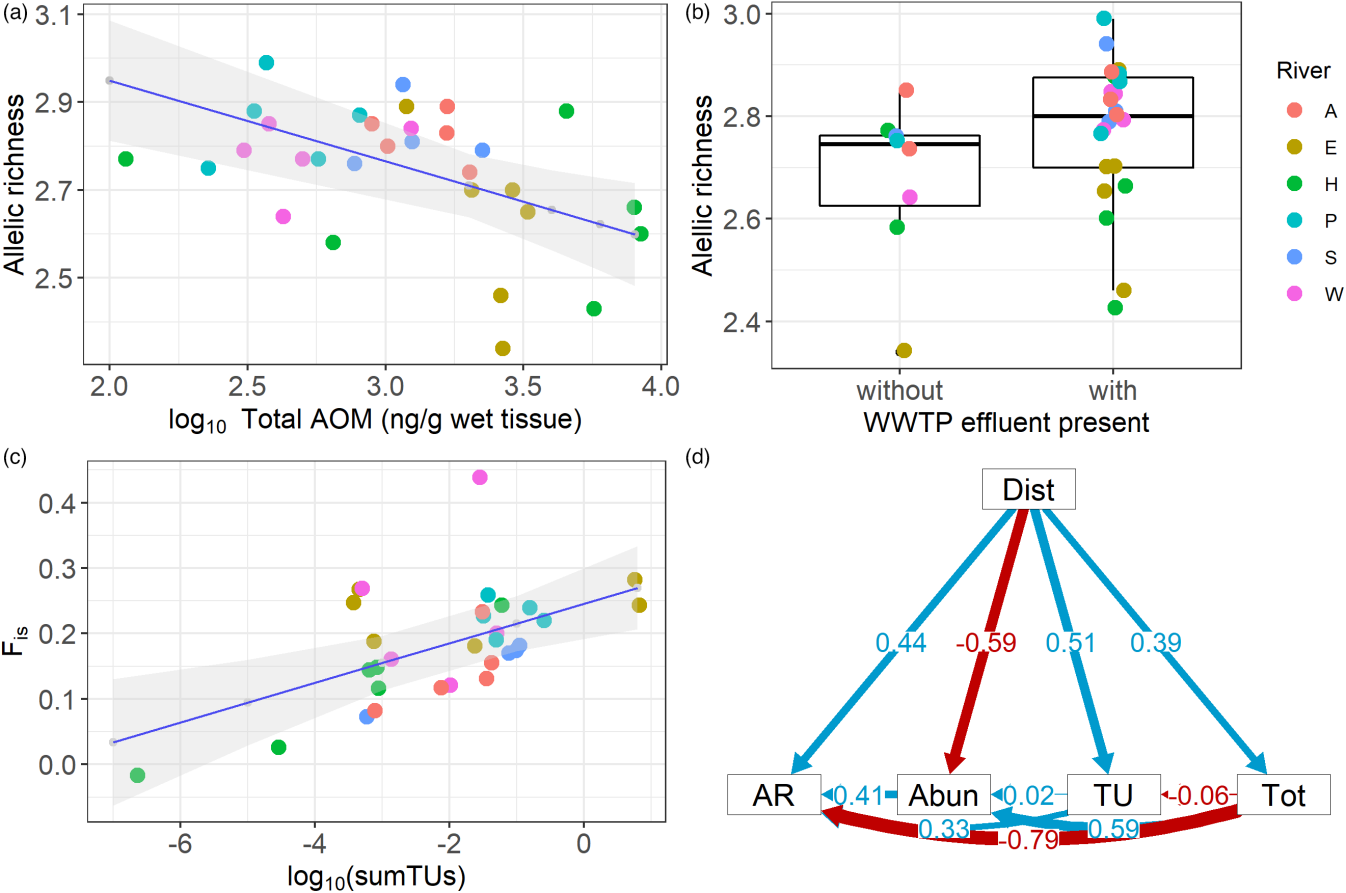
Relationship of *Gammarus pulex* genetic diversity parameters and AOM indices from the sampling sites at the investigated rivers. (a) Scatterplot based on a linear mixed‐effects model (regression line with 95% confidence interval shaded) of allelic richness values for *G*. *pulex* and the total AOM tissue concentration at the respective site. Circles represent the sampling sites, colors represent different rivers (A—the Altenau River, E—the Eine River, H—the Holtemme River, P—the Parthe River, S—the Saale River, and W—the Wipper River). (b) Box plots of allelic richness values of *G*. *pulex* from sampling sites upstream (without) or downstream (with) of a WWTP effluent. (c) Scatterplot based on a linear mixed‐effects model (regression line with 95% confidence interval shaded) of inbreeding coefficient (*F*_is_) values and toxic units (log_10_(sumTUs)). Circles represent the sampling sites, colors represent different rivers (refer to “A”). (d) Structural equation model graphical output indicating the relationship of site‐specific parameters allelic richness (AR), total concentration of AOM in *G*. *pulex* tissue (Tot), distance from the source (Dist), and intermediate parameters of TUs (TU) and *G*. *pulex* abundance (Abun). Blue arrows represent a positive relationship and red arrows a negative relationship of two parameters. The width of the arrows, together with the indicated values, represents the magnitude of the indicated relationship

Cluster values K with the highest probability that resulted from structure analysis of *G*. *pulex* genotypes were K = 2, K = 3, and K = 6 (Figure S4). For K=6, each cluster belonged to a specific river (Figure 3d). Some individuals with a genotype membership of another than the predominant cluster in a respective river were found particularly at sites E1, E6, H6, W1, and W2. The *F*_st_ values corresponded to the result of the genetic structure analysis with values significantly different from zero for all pairwise comparisons of rivers. Genetic differentiations were observed to be largest between *G*. *pulex* from the Saale River and the Holtemme River, from the Saale River and the Eine River, and from the Eine River and the Altenau River (Figure 3a). These pairwise comparisons indicated a subpopulation structure between sites with lower *F*_st_ values (*F*_st_ <0.1 in Holtemme:Eine and Altenau:Parthe:Wipper), consistent with K = 3 from the structure analysis (Figure 4).

The genetic differentiation of *G*. *pulex* within each river was lower than among rivers. *F*_st_ was highest with 0.079 for *G*. *pulex* from sites S1 and S4 (Saale River), followed by 0.075 for *G*. *pulex* from sites E1 and E6 (Eine River) and 0.055 for *G*. *pulex* from sites W1 and W6 (Wipper River; Table S10). The highest *F*_st_ values were detected when comparing *G*. *pulex* genotypes from the most upstream and the most downstream sites in the rivers (Table S10). Of 81 pairwise *F*_st_ values for comparisons within rivers, 35 comparisons showed significant differences from 0 (Table S10). A significant positive relationship was detected between *F*_st_ values and waterway distances regionally (among all sites; *r* = 0.527, Mantel *p* < 0.001; Figure 3b) and locally (within single rivers; Figure S2), except for the Holtemme River for which the *F*_st_ value–waterway distance relationship was nonsignificant (*p* > 0.05). Surprisingly high *F*_st_ values were detected for two sites located close to each other (i.e., P1 and P2; Figure S2c) and for two sites with similar pollution and toxicity patterns (i.e., H4 and H6; Figure S2d). At the regional scale and accounting for the effect of waterway distances, genetic differentiation increased with differences in total AOM levels (*r* = 0.173, Mantel *p* < 0.01). Within rivers, partial Mantel tests were not significant, except for the Altenau River. Genetic differentiation did not increase with differences in TUs neither regionally nor locally (Mantel *p* > 0.3).

### 3.4 Relationship between AOM and *Gammarus pulex* genetic diversity indices

The association between *G*. *pulex* genetic diversity parameters and AOM concentrations detected in *G*. *pulex* tissue was indicated by LMEs. A significant contribution of fixed effects to LME was indicated for 4 of the 5 analyzed model indicators, including allelic richness, F_is_, abundance, and N_e_ (Table S11). The distribution of private alleles could not be explained by any linear effect across analyzed rivers. According to the AICc and log‐likelihood values, models without explanatory variables described genetic diversity parameters equally well as the models with defined fixed effects, suggesting that other than the here considered fixed effects influenced the assessed genetic parameters (Table S11).

Three of the analyzed fixed effects, total AOM concentration, TUs, and the presence of WWTP effluent, showed a significant correlation with the analyzed indicators (*p* < 0.05). The presence of WWTP effluent and total concentration of AOM best‐described changes in allelic richness in *G*. *pulex* (Table S11). The presence of WWTP effluent showed a positive relationship to the allelic richness across the six rivers, yet, the relationship was not confirmed by analysis for each river individually (Table 1, Figure 4b). In contrast to the WWTP effluent, total AOM concentration was negatively correlated with the allelic richness at the studied sites (Table 1, Figure 4a). According to the LME regression, allelic richness was 12% lower at the highest measured AOM concentrations in comparison with the lowest concentrations of AOM. It was also up to 22% reduced at sites with increased levels of AOM in comparison to sites with low AOM pollution. The distribution of *F*_is_ from the analyzed sites was associated with TUs calculated from AOM detected in *G*. *pulex* (Table 1). The calculated TUs were positively related to the *F*_is_ rates (Table 1, Figure 4c), which, according to the model, spanned from *F*_is_ of 0.04 at low TU values to *F*_is_ of 0.36 at high TU values. The abundance of amphipods at sampling sites was best described by the WWTP effluent and total concentration of AOM (Table 1). The abundance of *G*. *pulex* was negatively correlated with WWTP effluents and positively correlated with the total concentration of AOM (Table 1). Abundances were lowest at sites with very high or very low TU of AOM in both river water and *G*. *pulex* tissue samples (e.g., at sites H1, W5, W6). Finally, the most informative fixed effect associated with the effective population size of *G*. *pulex* was the presence of WWTP effluent, with a positive but nonsignificant relationship (Table 1).

**TABLE 1:** Parameter values from LMEs for the analyzed genetic diversity indicators allelic richness, inbreeding coefficient (*F*_is_), abundance, effective population size (N_e_), and respective fixed effects

Indicator	Fixed effect	Estimate	SE	*t*‐Value	*p*‐Value
Allelic richness	Intercept	3.189	0.160	19.875	<0.001
	WWTP +	0.173	0.049	3.526	0.002
	log_10_ total AOM	−0.184	0.056	−3.308	0.003
F_is_	Intercept	0.313	0.062	5.089	<0.001
	TU_gam_	0.066	0.029	2.282	0.032
Abundance	Intercept	−0.409	0.562	−0.728	0.474
	WWTP +	−0.462	0.166	−2.772	0.012
	log_10_ total AOM	0.775	0.194	3.985	0.001
log_10_ N_e_	Intercept	1.722	0.141	12.240	<0.001
	WWTP +	0.293	0.163	1.794	0.0854

Note

The parameters intercept estimation (Estimate), standard error (SE), *t*‐values, and *p*‐values are indicated. Fixed effect indicate a wastewater treatment plant effluent source upstream of the sampling sites (WWTP +), log_10_ of the total concentration of the detected AOM in *G*. *pulex* tissues (log_10_ total AOM); toxic unit values calculated from AOM concentrations detected in *G*. *pulex* tissues (TU_gam_).

The relationship between the water concentrations of total AOM and allelic richness was confirmed by the global SEM. The model combining allelic richness, total AOM water concentration, and distance to the source with intermediate variables indicated the strongest negative relation between total AOM and allelic richness (−0.79, *p* < 0.02) and a positive relationship between distance to the river source and allelic richness (Figure 4d). The negative relationship between total AOM and allelic richness was also detected in models assessing parameters of single rivers. The effects of total AOM on allelic richness spanned from the value of −0.15 in the Wipper River to the value of −0.94 in the Saale River (Figure S5), yet ranged from significant (rivers Eine, Wipper) to nonsignificant (rivers Altenau, Holtemme, Parthe, Saale), indicating river‐specific patterns (Table S12).

## 4 DISCUSSION

In this study, the association of genetic diversity of the freshwater amphipod species *G*. *pulex*, common in likewise polluted and pristine sections of rivers in central Europe, and AOM levels in the habitats of *G*. *pulex* was investigated. The population genetic structure of *G*. *pulex* in the studied region corresponded to river catchments and showed a weak correlation with the respective AOM contamination across rivers. However, genetic diversity parameters indicated a significant trend of reduced allelic richness and enhanced inbreeding rates of *G*. *pulex* from sites with increased levels of AOM.

### 4.1 The genetic structure of *Gammarus pulex* relates to the connectivity among sites

According to the genetic structure analysis performed here, populations of *G*. *pulex* in the studied region are strongly defined by the within‐river connectivity and the geographic distance between studied sites. The river‐related genetic structure of the examined species is in line with previous studies demonstrating the importance of riverine network and species colonization history for the genetic structure of amphipods in rivers (Weiss & Leese, 2016; Westram et al., 2013). Indeed, populations with a specific genetic structure of the amphipod *G*. *fossarum* were found to be confined by different river catchments based on neutral loci (Westram et al., 2013). Such river‐related genetic structure can be maintained by a high migration rate within a river that can compensate for selective drivers including local environmental stressors, such as increased pollution, large temperature oscillations, food scarcity, or increased competition (Lenormand, 2002).

In addition to river‐related *G*. *pulex* population structure, sites within rivers with significant differentiation levels were detected. The within‐river differentiation largely followed the isolation by distance pattern, yet it was high among some sites within rivers. For instance, reference site E1 and the other sites in the Eine River showed significant differentiation. Such differentiation is, due to reduced allelic richness at the reference site, likely not the result of the increased AOM input downstream of this site. Another factor contributing to isolation and reduced genetic diversity of *G*. *pulex* at the site E1 could be drought, as some parts of the stream dry out in summer. Finally, organic compounds released from the WWTP downstream of the site E1 could contribute to the absence of *G*. *pulex* directly downstream of the WWTP and to the spatial and genetic isolation of *G*. *pulex* at site E1. The other sites with significant differentiation were, in contrast to the sites in the Eine River, spatially distant from each other, or did not indicate the difference in pollution patterns.

Genetic differentiation has been found to be comparatively large not only among *G*. *pulex* from distant rivers but also in some *G*. *pulex* populations from spatially proximate rivers (Figure 3a). As shown by previous studies, genetic differentiation within *Gammarus* populations living at sites close to each other can be significant (Weiss & Leese, 2016; Zickovich & Bohonak, 2007). Some of this differentiation may be associated with increased levels of AOM as shown by the partial Mantel test (see Result Section 3.3). However, large genetic differentiation rates between populations were shown to be associated with migration barriers, local and seasonal gene flow bottlenecks, and drift (Reid et al., 2016; Weston et al., 2013). In *G*. *pulex* studied here, historic migration events could, on the one hand, explain the genetic similarity of *G*. *pulex* in geographically more distant rivers (e.g., Parthe and Altenau) and, on the other hand, the significant genetic divergence among *G*. *pulex* in geographically proximate rivers (e.g., rivers Eine and Wipper; Alp et al., 2012; Weiss & Leese, 2016). Moreover, the dispersal of amphipods by birds may contribute to gene flow to remote, hydrologically little connected sites, leading to low genetic differentiation among sites (Figuerola & Green, 2002; Rachalewski et al., 2013). Such events could promote the introduction of novel genotypes to the established populations in the river that could be reflected by differences in genetic membership of some individuals within a river.

### 4.2 Genetic diversity of *Gammarus pulex* at sites with AOM contamination

The negative relationship between allelic richness and total concentration of AOM (Figure 4a) and a positive relationship between inbreeding rates of *G*. *pulex* and TUs (Figure 4c) determined for the studied sites confirms the hypothesis that *G*. *pulex* from AOM‐polluted habitats exhibits reduced genetic diversity. The reduced genetic diversity in *G*. *pulex* at sites with comparatively high levels of AOM can be attributed to an increased probability of genetic drift and loss of rare alleles (Hoffmann & Willi, 2008). The effects of genetic drift on populations exposed to AOM are even more likely when considering many other environmental and biological stressors, such as high summer temperatures that facilitate the susceptibility of organisms to toxicants (Brans et al., 2021). Similar to the findings presented here, reduced genetic diversity was found in populations of *Daphnia magna* in ponds with increased levels of AOM, suggesting their selective pressure (Coors et al., 2009). In exposed *G*. *pulex*, the selective pressure of AOM with toxic potential might promote genotypes beneficial in toxic environments. However, selective effects of AOM might be masked by gene flow between sites or the examined microsatellites may not be associated with genes under selection, resulting in no significant genetic change in the studied populations. Still, migration does apparently not proceed at a rate that would compensate for low allelic richness at sites with increased AOM levels. In addition, increased inbreeding rates in *G*. *pulex* strongly relate to estimated toxic levels of AOM. Increased inbreeding can enhance the effects of AOM in *G*. *pulex*, as it was shown to affect survival, reproduction, resistance to disease and predation, and susceptibility to environmental stress (Keller & Waller, 2002). These effects, resulting in reduced population fitness, are especially likely to occur in populations with a strong competition between males (Meagher et al., 2000). To prevent negative effects and retain a low inbreeding rate, inbreeding avoidance mechanisms exist (Pusey & Wolf, 1996), yet these appear to be ineffective in *G*. *pulex* living in the more polluted river sections.

In addition to the findings of decreased allelic richness and high inbreeding at sites with increased levels of AOM, allelic richness was increased at the sites downstream of WWTP effluent discharges (Figure 4b). This contrasts with the finding of reduced allelic richness downstream of the main pollution sources and is mainly due to comparatively low AOM levels and high allelic richness detected at multiple sites downstream of the WWTP effluents (e.g., A4, E4, E6, P3, W3, W4). These results might reflect the complexity of the environmental conditions co‐affecting the genetic diversity of *G*. *pulex* at the sites downstream of the WWTP. The effects of AOM may be altered by nutrients entering the river *via* the WWTP effluents. In fact, the abundance of *G*. *pulex* was particularly high at some downstream sites (e.g., A3–A5, H3–H6), which could be related to high nutrient levels from WWTP effluents and thus the high abundance of food for *G*. *pulex*. The higher genetic diversity of *G*. *pulex* at sites WWTP effluents may thus be due to the abundance of *G*. *pulex* at these sites that is comparatively high because of the increased availability of food. Furthermore, comparatively high genetic diversity at downstream sampling sites may be due to the comparatively long distance to the river source. According to the LME, distance from the source was not an important parameter for increased allelic richness within a river (see Table S11). Yet, the allelic richness and private allele values were highest at the most downstream sampling sites, which may be due to their proximity to the river confluence and therefore enhanced gene flow from other *G*. *pulex* populations (Alther et al., 2021).

### 4.3 AOM compounds with the potential to alter the genetic diversity of *Gammarus pulex*

AOM can alter the genetic diversity of exposed species by causing mutagenic effects by exerting direct selective pressure or by affecting a species’ gene pool through nonselective effects (Bickham, 2011). Mutagenic effects may lead to an increased genetic diversity at sites with mutagenic and genotoxic compounds present (Theodorakis et al., 2001). At sites E6 (Eine River) and W2 (Wipper River), where mutagenic or genotoxic compounds were found, allelic richness in *G*. *pulex* was highest; contrariwise, at site P1, where one compound with mutagenic potential was found, allelic richness was lowest in *G*. *pulex* across sites in the Parthe River. Thus, additional research would be necessary to reveal whether mutagenic and genotoxic AOM significantly contribute to changes in allelic richness in a multiple‐stressors context.

From the detected AOM, insecticides can be expected to exert selective pressure because of their high toxic potential. Particularly, the detected insecticides fipronil, imidacloprid, thiacloprid, clothianidin, and acetamiprid were shown to cause adverse effects in *G*. *pulex* by hindering mobility and feeding (Englert et al., 2017) and, thus, select for tolerant individuals. Concentrations of insecticides in the same toxicity range as detected at sites with high toxic potentials (such as at e.g., E3, E6, P4, P5) were shown to reduce genetic diversity and promote specific genotypes adapted to the particular insecticide exposure, for example, for pyrethroid exposure in *H. azteca* (Weston et al., 2013). An increased tolerance of *G*. *pulex* to these AOM may improve *G*. *pulex* performance; however, selection for tolerant individuals likely results in the observed inbreeding and a decrease in allelic richness at these sites. In addition, *G*. *pulex* at sites with high AOM levels are exposed to other pesticides possibly exerting indirect selective pressures. These include herbicides, fungicides, and biocides, which affect freshwater communities by reducing the quantity and diversity of periphyton, freshwater plants (e.g., MCPA, DEET, and pendimethalin), or fungi (carbendazim) that *G*. *pulex* feeds upon. It may be assumed that these indirect effects lead to an increase in intraspecific competition and genetic drift; however, there is no experimental evidence yet for the consequences of these indirect effects. Furthermore, effects caused by freely dissolved AOM in water and by AOM in *G*. *pulex* tissue should be compared in future studies, as the detected compound concentrations and exposure duration often largely differ. For instance, AOM may persist over long time periods in the tissue of *G*. *pulex*, while the exposure of *G*. *pulex* to some freely dissolved compounds would only occur at the time of events such as the release of wastewater from the WWTP.

For many AOMs detected at sites with reduced genetic diversity of *G*. *pulex*, no information on acute toxicity for *G*. *pulex* is available (see Table S2). These AOMs that were detected at exceptionally high concentrations, often exceeding 100 µg/L, included the pharmaceuticals diclofenac, theophylline, valsartan, hydrochlorothiazide, 4‐aminoantipyrine, the industrial chemicals 1H‐benzotriazole, melamine, guanylurea, 2‐benzothiazolesulfonic acid, 7‐diethylamino‐4‐methylcoumarin, and tris(1‐chloro‐2‐propyl)phosphate and the food additive triethylcitrate. Some of these AOMs can contribute to alterations of a species’ genetic diversity by increasing selective pressure or altering the inheritance of alleles by affecting species reproduction or behavior. For example, diclofenac may exert selective pressure on and increase genetic drift of *G*. *pulex,* as it was shown to cause reduced survival of macroinvertebrates, including amphipods, when in a mixture with other AOM (Miller et al., 2015). In addition to the acute toxic effects of AOM, endocrine disruptors found in *Gammarus* amphipods were shown to alter the male‐female ratio, influence reproductive success, and alter population size and allele frequencies in the exposed populations (Gross et al., 2001; Watts et al., 2002). In the current study, 7‐diethylamino‐4‐methylcoumarin, a driver of antiandrogenic effects in fish, was for the first time detected at high concentrations in the Eine River and confirmed for the Holtemme river (Muschket et al., 2018; Švara et al., 2021). The effects of this coumarin in amphipods remain unknown. However, it could importantly contribute to a complex pattern of allele frequency change, as antiandrogenic effects were indicated to contribute to genetic diversity change by altering sexual behavior in fish (Alves da Silva et al., 2018).

### 4.4 Ecological relevance of AOM effects in *Gammarus pulex*

Altered genetic diversity of a species can have significant consequences for the species’ ecological performance. Reduced genetic diversity within populations can impact the species’ abundance and thus its ecological function, interspecific competition, and the species’ ability to recover from disturbance (Randall Hughes et al., 2008). In the current study, such low abundance accompanied by a small effective population size of *G*. *pulex* was detected at sites with extremely high, potentially acutely toxic levels of AOM (e.g., W5, W6, see Table S8). *Gammarus pulex* at these sites may, due to reduced genetic diversity, show disrupted ecological performance, which was found to be associated with decreases in survival, body size, and reproduction (Aguirre‐Gutierrez et al., 2015).

Reduced abundance and genetic diversity of a species may cause changes in interspecific competition and species community structure. In the Eine River, *G*. *pulex* was found to co‐occur with *Gammarus roeselii* Gervais, 1835. *Gammarus pulex* can survive in sympatry with other amphipod species (Altermatt et al., 2019); however, the abundance of *G*. *pulex* was reduced at sites E2–E6 inhabited by both species. The allelic richness within *G*. *pulex* was not reduced at these sites. In contrast, comparatively low genetic richness but high abundance of *G*. *pulex* was seen at site E1 (Eine River), where no *G*. *roeselii* were found, indicating a genetic bottleneck for *G*. *pulex*. Comparable abundance patterns were shown for amphipod species that occupy similar ecological niches in streams, suggesting that colonizing history majorly influences species composition (Little & Altermatt, 2018).

In addition to interspecific competition, low genetic diversity at sites with comparably high AOM levels can affect the ability of *G*. *pulex* to respond to environmental changes in the long run. In populations with critically reduced genetic diversity the effects of environmental stress factors may be more pronounced, increasing species mortality and threatening its survival (Pearman & Garner, 2005). Moreover, chronic exposure to toxic AOM was shown to increase the susceptibility of *G*. *pulex* to additional acute stress (Švara et al., 2021). Thus, a combination of multiple environmental stressors, such as temperature extremes or increased parasitism, in parallel with increased levels of AOM, poses an increased adverse risk for *G*. *pulex* populations exhibiting low genetic diversity.

In conclusion, our results indicate that AOM contamination of rivers and streams can significantly shape the population genetic diversity of *G*. *pulex*. A decline in the genetic diversity of the species may lead to decreased species robustness to environmental stress that, in the long run, can affect the survival of this keystone species and enhance the risk of the loss of its ecosystem function.

## CONFLICT OF INTEREST

The authors declare no conflict of interest.

## Supporting information

Supplementary Material

Table S2

Table S3

Table S4

Table S5

## Data Availability

Data on measured AOM concentrations and microsatellite genotypes are available on Dryad (https://doi.org/10.5061/dryad.zw3r2288p). Sequencing data are available in GenBank.
