# Supplementary material for: Reduced genetic diversity of freshwater amphipods in rivers with increased levels of anthropogenic organic micropollutants
Source: Evol Appl. 2022 May 10;15(6):976–91. doi: 10.1111/eva.13387 (PMC9234654; doi:10.1111/eva.13387)
Supplement: Supplementary file 1 — Supplementary Material [file EVA-15-976-s001.docx]

**Supplementary Information (SI)**

**Reduced genetic diversity of freshwater amphipods in rivers with increased levels of anthropogenic organic micropollutants**

**Supplementary Text**

**Material and Methods**

S1 LC-HRMS sample preparation and analysis

S1.1. Water sample analysis

The pH of 1 mL water sample aliquots was adjusted to 3.5 by adding 10 µL of 2 M ammonium formate buffer to roughly match that of the LC eluent (0.1% formic acid, pH 2.6); 25 µL of an internal standard mixture containing 40 isotope-labelled compounds (40 ng/mL of each compound) and 25 µL of methanol were added. Matrix-matched calibration standards were prepared in the same way by spiking 1 mL water sample aliquots from a pristine stream (Wormsgraben, upper Harz Mountains, Germany) with the target analytes at concentrations from 1 to 5000 ng/L.

S1.2. Body burden analysis

*Gammarus pulex* tissue samples were extracted using the QuEChERS (Quick, Easy, Cheap, Effective, Rugged and Safe) method according to Inostroza et al. (2016) Accordingly, 900 mg of amphipod tissue was homogenized in a mixture with 2 mL water, 2 mL acetonitrile, and 1 mL hexane using an Ultra-Turrax T-25 (IKA) for ca. 60 s followed by vortexing of the suspension for 60 s. Subsequently, 800 mg anhydrous MgSO_4_ and 200 mg NaCl were added, the samples were vortexed again and centrifuged (4000× *g*) for 5 minutes. The supernatant was transferred to centrifugation tubes containing 400 mg anhydrous MgSO_4_ and 50 mg primary-secondary amine. The suspension was vortexed and centrifuged. The supernatant was moved into an evaporation vial and dried under the nitrogen stream at 23 °C. Finally, 50 µL of internal standard solution and 450 µL methanol (final level 100 ng/mL) were added. Method-matched calibration standards were prepared by spiking target analyte solutions into 2 mL LC-MS grade water, which were processed the same way as the samples, corresponding to final levels of 0.1–200 ng/g in vial.

S1.3. LC-HRMS analysis

Water samples and *Gammarus* extracts were analyzed by LC-HRMS using a Thermo Ultimate 3000 LC system (consisting of a ternary pump, autosampler and column oven) coupled to a quadrupole-orbitrap instrument (Thermo QExactive Plus) via a heated electrospray ionization source. LC separation was done on a Kinetex C18 EVO column (50 × 2.1 mm, 2.6 µm particle size) using a gradient elution with 0.1% of formic acid (eluent A) and methanol containing 0.1% of formic acid (eluent B) at a flow rate of 300 µL/min. After 1 min of 5% B, the fraction of B was linearly increased to 100% within 12 min and 100% B were kept for 11 min. The eluent flow was diverted to waste and the column was rinsed for 2 min using a mixture of isopropanol + acetone 50:50 / eluent B / eluent A (85% / 10% / 5%) to remove hydrophobic matrix constituents from the column. Finally, the column was re-equilibrated to initial conditions for 5.7 min. The injection volume was 5 µL for *Gammarus* extracts and 100 µL for water samples and the column was operated at 40°C. The heated ESI source and the transfer capillary were both operated at 300°C, the spray voltage was 3.8 kV (positive mode) or 3.5 kV (neg. mode), the sheath gas flow rate was 45 a.u. and the auxiliary gas flow rate 1 a.u. Separate runs were conducted in positive and negative ion mode combining a full scan experiment (100–1000 m/z) at a nominal resolving power of 70,000 (referenced to m/z 200) and data-independent MS/MS experiments at a nominal resolving power of 35,000. For the latter, we acquired the data using broad isolation windows of about 50 mass units (i.e., m/z ranges 97–147, 144–194, 191–241, 238–288, 285–335, 332–382, 379-429, 426–476) and 280 Th (i.e., m/z ranges 460–740, 730–1010), respectively.

S1.4 Compounds quantification and estimation of toxic effects in *G. pulex*

Raw data from the LC-HRMS analysis were converted into .mzML format using ProteoWizard v3.0.18265. The peak list for each batch was generated by MZmine v2.32 (Pluskal et al., 2010), with settings set as suggested in Beckers et al. (2020) and annotated from a list of 534 target compounds. The extracted list was corrected for blanks according to equation 1 (see below). Signals below the threshold in the samples were removed, following by an intensity cut-off of peaks with intensity of 5,000 for negative mode and under 50,000 for positive mode batches, in order to remove the noise created by gap filling. The data containing compounds from negative and positive mode batches was assembled in a table.

Equation 1:

$$I_{thres}=\mu\left( I_{Blk} \right)+2* \sigma(I_{Blk})$$

I_thres_ = intensity threshold

μ(I_Blk_) = mean of peak intensities in blanks;

σ(I_Blk_) = standard deviation of peak intensities in blanks

S2 Polymerase chain reactions, sequencing and genotyping information

COI PCR and sequencing reactions

50 µL PCR reactions contained 2.5 µL of 10 mM dNTPs, 10 µL 5X Green GoTaq Flexi Buffer (Promega), 4 µL of 25 mM MgCl_2_, 1 µL of GoTaq DNA polymerase and 22.5 µL of deionized Water. Each primer (Supp. 2) was diluted from the stock solution to 10 µM and 2.5 µL were added to the PCR reaction. Finally, 5 µL of DNA template with concentration between 40-80 ng/µL was added to the mix. The polymerase chain reaction (PCR) cycling setting was set to initial activation step of 2 min at 95°C, following by 34 cycles of 1 min denaturation at 95°C, 45 s annealing at 51°C and 1 min elongation at 72°C. The reaction was terminated after the final elongation of 5 min at 72°C. The PCR products were checked on the agarose gel and cleaned using GeneJET PCR Purification Kit (ThermoFisher Scientific) following the kit instructions. The 10 µL sequencing reaction of 150-250 ng of DNA with 1µL of the sequencing primer, Big dye mix (Thermo Fisher Scientific) and 5x sequencing buffer respectively was prepared for each PCR product. With the program as following: 1 min at 96°C, 30 cycles of 10 s at 96°C, 5 s at 50°C and 4 min at 60°C. The products were purified by ethanol/EDTA (ethylenediaminetetraacetic acid) precipitation protocol (Applied Biosystems, 2010), and diluted in 10 µL HiDi formamide (Thermo Fisher Scientific). The samples were separated on an ABI Prism 3130XL Genetic Analyzer (Applied Biosystems).

S3 COI sequence analyses and visualization

COI sequence reads were assembled and edited in Sequencher 5.4.5, with gaps coded as (-). Sequence contigs and, as reference, sequences of *G. pulex* from European rivers acquired from National Center for Biotechnology Information (NCBI) (KT075230.1_G_pulex_E, KT075232.1_G_pulex_D, KT075256.1_G_pulex_C, KF521835.1_G_fossarum were aligned using ClustalW in MEGA7 (Kumar et al., 2008) with default settings for alignment. Based on the alignment, maximum likelihood tree with best fitting Tamura 3-parameter model and nearest-neighbor-interchange tree Inference method (Tamura et al., 2011) were constructed. Confidence in the obtained topology was assessed by bootstrapping the dataset 1000 times. The phylogenetic tree was visualized with FigTree v1.4.3 (http://tree.bio.ed.ac.uk/software/figtree/).

**Supplementary Figures:**


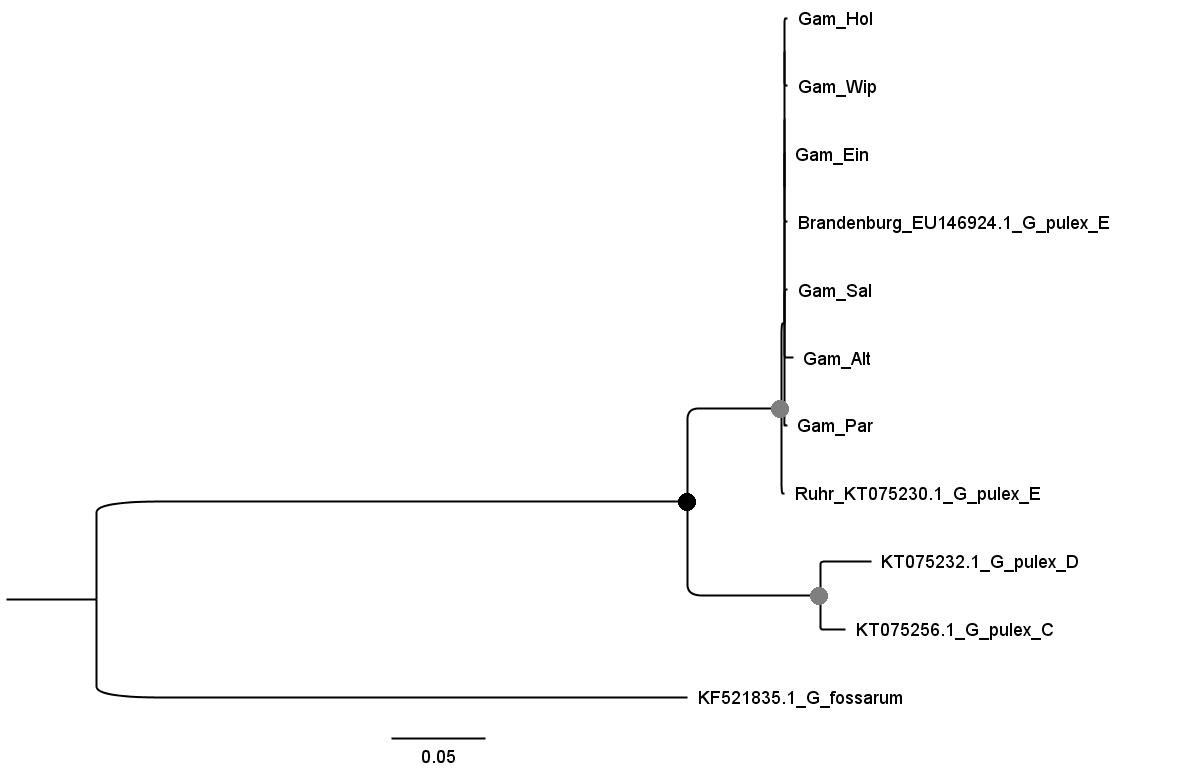


Supplementary figure S1. Phylogenetic position of amphipods studied from six rivers in central Germany. The tree was constructed based on COI maximum likelihood comparison of the obtained *G. pulex* sequences indicating genotypes from six rivers. Black dot denotes the clade with bootstrap values of > 99, while grey dots denote bootstrap values of > 95. The sequences from the analysed rivers can be accessed in GenBank by the codes MN400976 (Gam_Hol), OL441362 (Gam_Wip), OL441361 (Gam_Ein), MN400975 (Gam_Sal), OL441360 (Gam_Alt), MN400977 (Gam_Par).

**
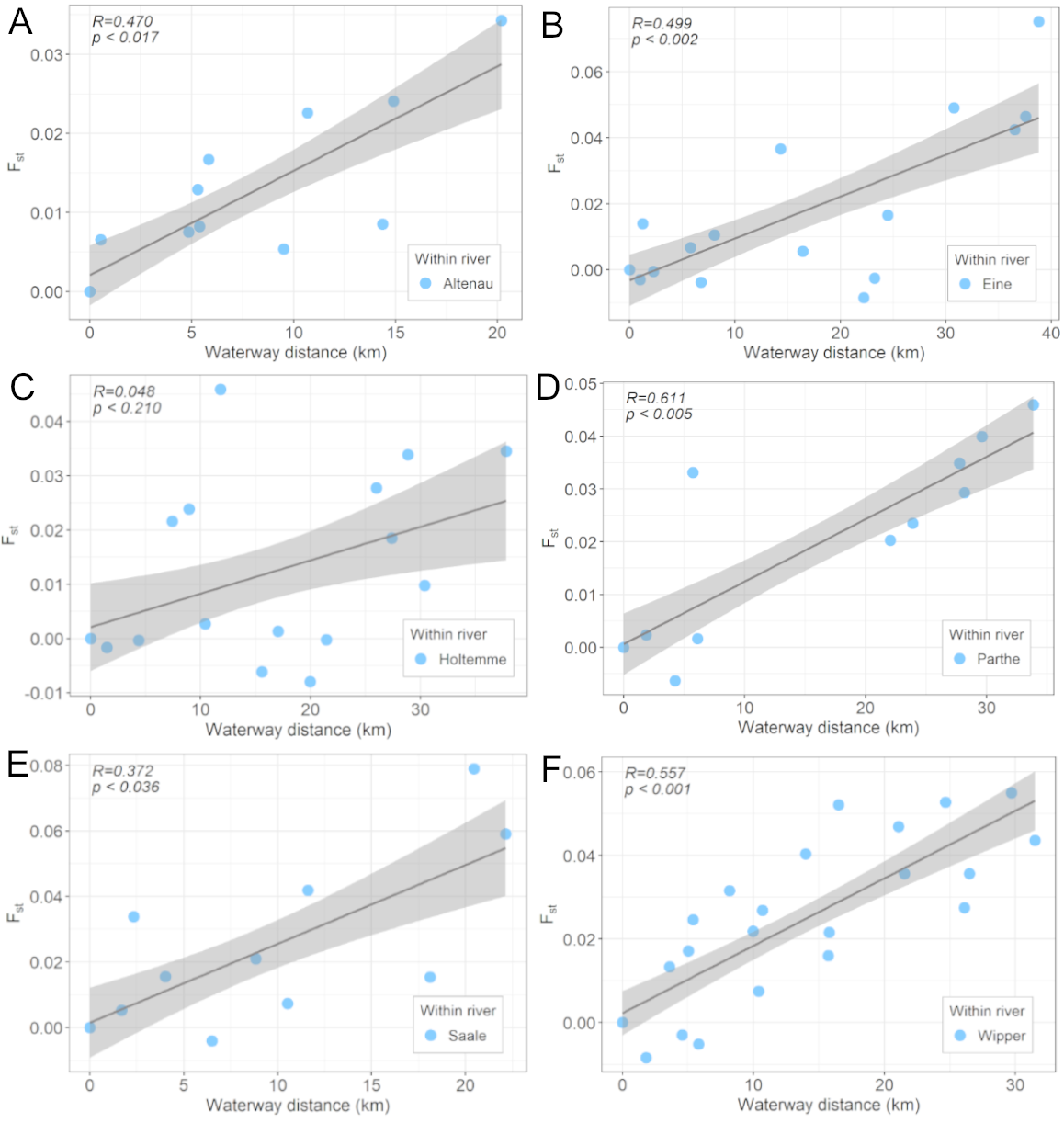
**

Supplementary figure S2. Mantel test of pairwise F_st_ values between every pair of sites within each river (A–F) with respective waterway distances (km). Grey line indicates linear regression of the Mantel test with a 95% confidence interval (shaded area).


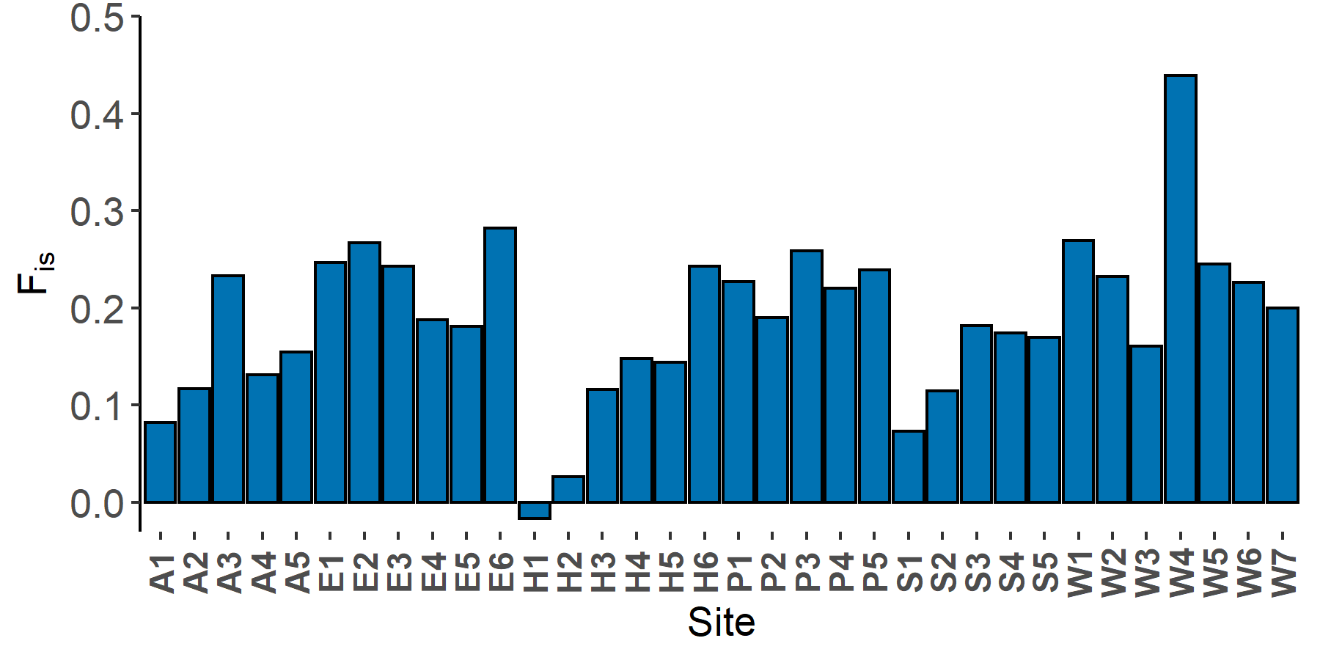


Supplementary figure S3. Inbreeding coefficient rates (F_is_) of amphipods samples from 34 sites along the Rivers in central Germany.


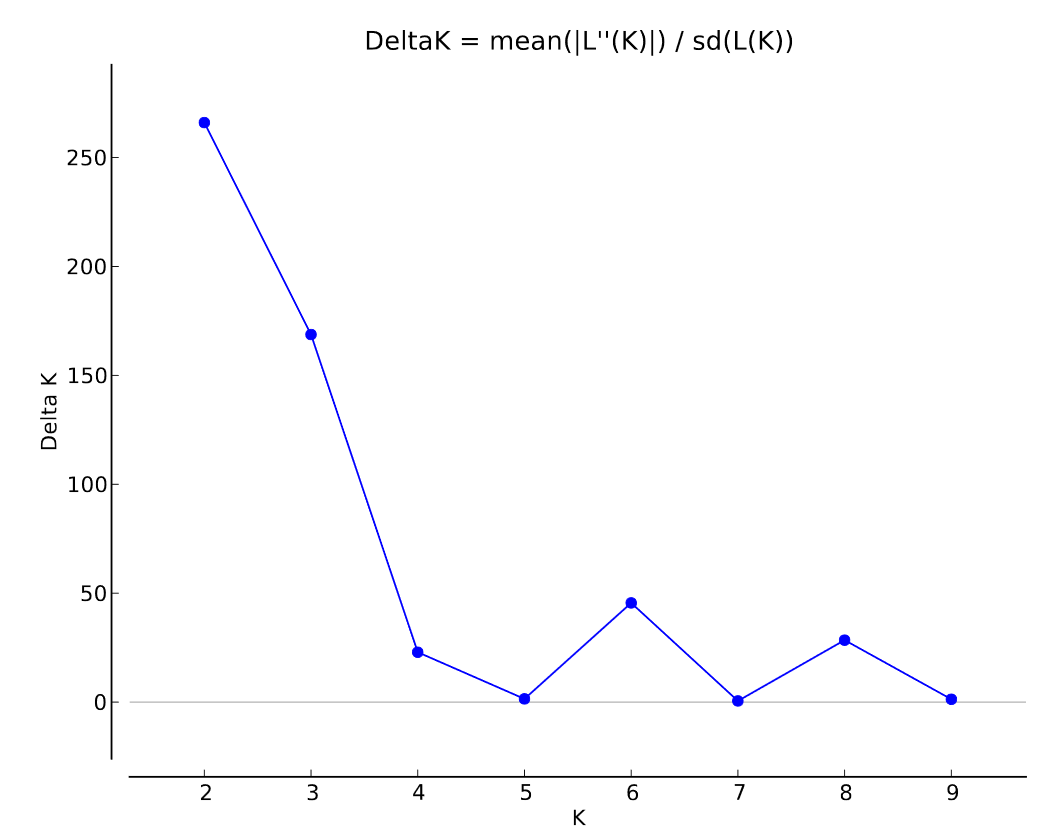


Supplementary figure S4. Delta K values for *G. pulex* from Structure Harvester. The identification of the most likely number of genetic clusters K for K 1–9 was done using the Evanno method based on 16 microsatellite loci.

**
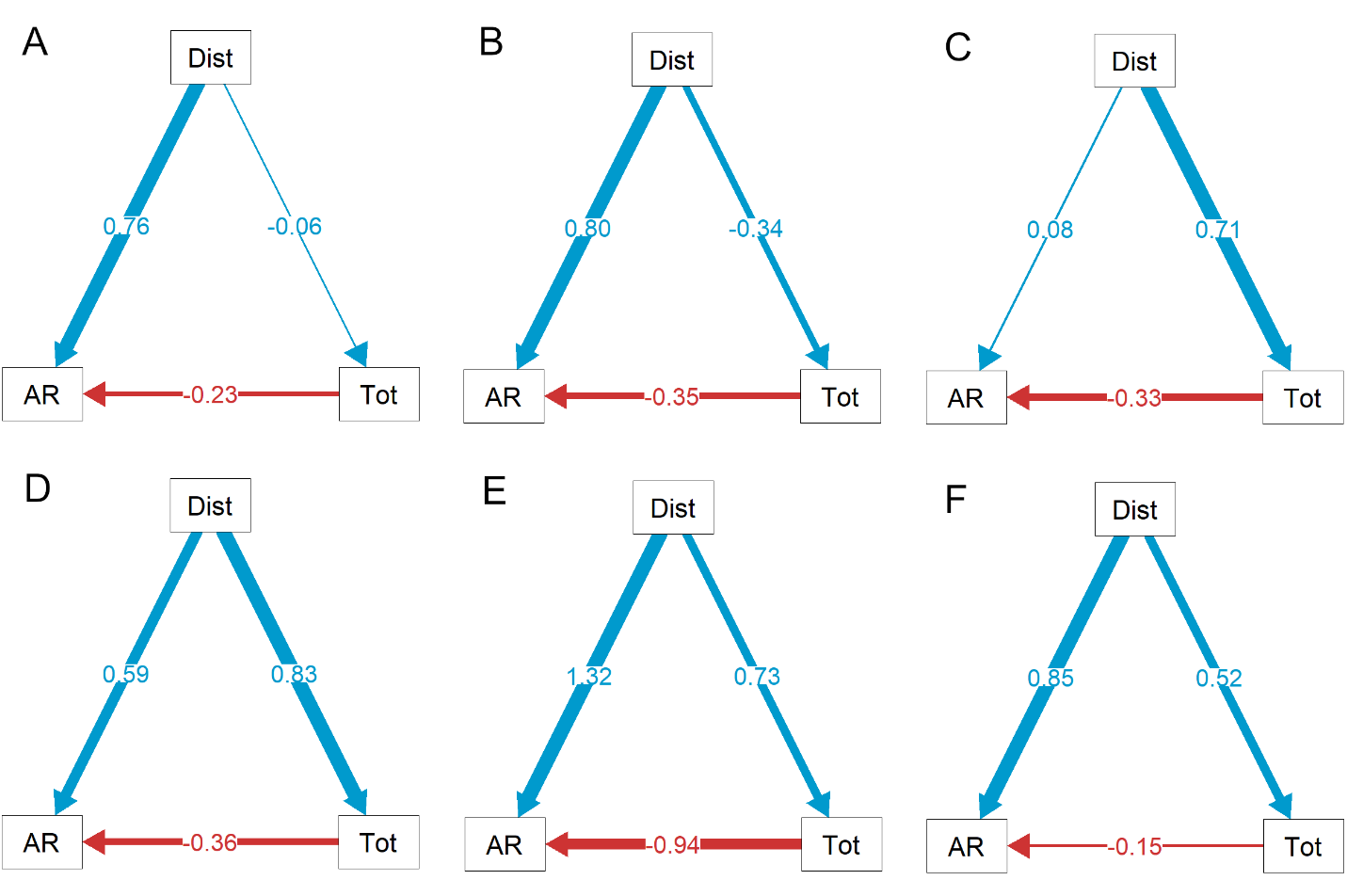
**

Supplementary figure S5. Output of the standard equation models for each river (Altenau (A), Eine (B), Holtemme (C), Parthe (D), Saale (E), Wipper (F)) comparing the relationship between allelic richness (AR), distance of the sampled site from the source (Dist), and total concentration of AOM (Tot) detected at each sampling site. Blue color denotes positive relationship and the red color denotes negative relationship. The arrows width is scaled according to the value of each relation.

**Supplementary Tables:**

Supplementary table S1. Studied sites at the rivers Altenau (A), Eine (E), Holtemme (H), Parthe (P), Saale (S), and Wipper (W). Sampling site codes, site names, sampling dates and times, sampling site coordinates, temperature, pH value, water oxygen concentration, conductivity, number of individuals per catch as a measure of abundance of *G. pulex* at sampling site, information of presence of WWTP effluents upstream of the sampling sites (WWTP upstream), and the number of *G. pulex* sampled for DNA analysis at each site are listed.

| Site code | Site name | Sampling date and time | Coordinates (Lat, Long) | T (°C) | pH | O_2_ (mg/L) | | Conductivity (µS/cm) | Abun.  (per catch) | WWTP upstream | Sampled *G. pulex* (DNA) |
| --- | --- | --- | --- | --- | --- | --- | --- | --- | --- | --- | --- |
| A1 | Altenau. Upsteam of Schöppenstedt | 11.11.2019  14:00 | 52.157923; 10.820882 | 6.9 | 7.31 | 10.9 | 953 | | 100 | No | 30 |
| A2 | Altenau. Schöppenstedt. Upstream of WWTP | 11.11.2019  13:00 | 52.141719; 10.762080 | 4.9 | 7.48 | 12.91 | 1059 | | 60 | No | 30 |
| A3 | Altenau. Schöppenstedt. Downstream of WWTP | 11.11.2019  12:00 | 52.143531; 10.757041 | 6.3 | 7.52 | 11.82 | 1049 | | 120 | Yes | 30 |
| A4 | Altenau. Weferlingen | 11.11.2019  11:00 | 52.151367; 10.688176 | 4.5 | 7.89 | 14.67 | 1197 | | 150 | Yes | 30 |
| A5 | Altenau. Klein Denkte | 11.11.2019  10:00 | 52.140427; 10.575466 | 3.7 | 7.84 | 12.44 | 1970 | | 100 | Yes | 30 |
| E1 | Eine. Upstream of WWTP Schielo | 9.1.19  8:00 | 51.616644; 11.211005 | 4.4 | 7.38 | 12.09 | 330 | | 50 | No | 30 |
| E1a* | Eine. Downstream of WWTP Schielo | 9.1.19  10:00 | 51.612240. 11.219577 | 4.9 | 7.82 | 12.82 | 578 | | 0 | Yes | 0 |
| E2 | Eine. Stangerode | 18.12.18  14:00 | 51.650788; 11.348645 | 3.1 | 8.22 | 13 | 557 | | 50 | Yes | 30 |
| E3 | Eine. Upstream of Aschersleben | 18.12.18  13:00 | 51.737861; 11.439389 | 3.9 | 8.23 | 13.34 | 708 | | 10 | Yes | 30 |
| E4 | Eine. Upstream of WWTP Aschersleben | 18.12.18  12:00 | 51.743211; 11.481198 | 4 | 8.22 | 13.48 | 662 | | 10 | Yes | 30 |
| E5 | Eine. Downstream of WWTP Aschersleben | 18.12.18  11:00 | 51.748178; 11.493240 | 3.9 | 8.08 | 12.67 | 761 | | 10 | Yes | 30 |
| E6 | Eine. Groß Schierstedt | 18.12.18  10:00 | 51.748462; 11.510467 | 3.6 | 8.13 | 12.8 | 724 | | 10 | Yes | 30 |
| H1 | Holtemme. Wernigerode | 20.7.2017  9:00 | 51.847054; 10.791327 | 9.8 | 7.21 | 11.66 | 214 | | 5 | No | 20 |
| H2 | Holtemme. Silstedt upstream of WWTP | 15.3.2017  17:00 | 51.865251; 10.853865 | 7.5 | 7.62 | 11.91 | 201 | | 50 | No | 20 |
| H3 | Holtemme Silstedt downstream of WWTP | 15.3.2017  14:30 | 51.867732; 10.873714 | 8.3 | 7.55 | 12.52 | 278 | | 200 | Yes | 20 |
| H4 | Holtemme. Upstream of WWTP Halberstadt | 16.3.2017  09:30 | 51.909947; 11.074887 | 5.2 | 8.53 | 12.16 | 316 | | 500 | Yes | 20 |
| H5 | Holtemme. Groß Quenstedt. | 16.3.2017  14:00 | 51.923896; 11.110006 | 9.1 | 8.26 | 12.92 | 426 | | 200 | Yes | 20 |
| H6 | Holtemme confluence Bode | 16.3.2017  16:30 | 51.963426; 11.182576 | 9.6 | 8.31 | 12.37 | 507 | | 5 | Yes | 20 |
| P1 | Parthe. Glasten | 10.10.2019  9:00 | 51.171061; 12.695489 | 10.5 | 7.06 | 7.8 | 701 | | 100 | No | 30 |
| P2 | Parthe. Grossbardau | 10.10.2019  10:00 | 51.211815; 12.703265 | 11.1 | 7.03 | 25.5 | 646 | | 50 | Yes | 30 |
| P3 | Parthe. Borsdorf, Downstream of WWTP | 10.10.2019  12:00 | 51.342064; 12.529571 | 11.3 | 7.55 | 4.87 | 555 | | 10 | Yes | 30 |
| P4 | Parthe. Borsdorf, Downstream of WWTP | 10.10.2019  14:00 | 51.355769. 12.532703 | 15.2 | 8.53 | 6.72 | 818 | | 20 | Yes | 30 |
| P5 | Parthe. Dewitz | 10.10.2019  15:00 | 51.381616. 12.526498 | 12.8 | 8.06 | 1.95 | 820 | | 50 | Yes | 30 |
| S1** | Saale. Kaulsdorf. | 12.6.2018  17:30 | 50.613133; 11.393243 | 12 | 8.5 | 11.7 | 449 | | 200 | No | 30 |
| S2 | Saale. Rudolstad, confluence with Schwarza | 12.6.2018  16:00 | 50.684319; 11.323392 | 17.7 | 8.3 | 9.65 | 358 | | 2 | Yes | 30 |
| S3 | Saale. Rudolstadt, upstream of WWTP | 12.6.2018  13:30 | 50.719146; 11.352061 | 14.8 | 7.8 | 10.95 | 486 | | 10 | Yes | 30 |
| S4 | Saale. Rudolstadt, at the WWTP | 12.6.2018  11:30 | 50.720117; 11.377208 | 13.3 | 7.5 | 9.6 | 700 | | 50 | Yes | 30 |
| S5 | Saale. Rudolstad, Downstream of WWTP | 12.6.2018  10:00 | 50.718542; 11.398322 | 14.8 | 7.4 | 9.41 | 520 | | 30 | Yes | 30 |
| W1*** | Wipper. Upstream of Wippra | 14.10.2019  9:00 | 51.569797; 11.252111 | 12.9 | 7.59 | 8.5 | 423 | | 50 | No | 30 |
| W2 | Wipper. Upstream of Friesdorf | 14.10.2019  10:00 | 51.585998; 11.292676 | 13.5 | 7.85 | 8.09 | 469 | | 20 | Yes | 30 |
| W3 | Wipper. Downstream of Bisenrode | 14.10.2019  11:00 | 51.604347; 11.404161 | 14.1 | 7.82 | 8.11 | 492 | | 20 | Yes | 30 |
| W4 | Wipper. Downstream of Vatterode | 14.10.2019  13:00 | 51.606634; 11.462897 | 14.4 | 7.78 | 8.07 | 538 | | 20 | Yes | 30 |
| W5 | Wipper. At the WWTP Grossörner | 14.10.2019  14:00 | 51.627493; 11.507825 | 16.9 | 8.38 | 8.66 | 587 | | 2 | Yes | 21 |
| W6 | Wipper. Before WWTP Hettestedt | 14.10.2019  15:00 | 51.655010; 11.520962 | 15.8 | 8.29 | 8.98 | 577 | | 1 | Yes | 10 |
| W7 | Wipper. After WWTP Hettestedt | 14.10.2019  17:00 | 51.665774; 11.537412 | 16.2 | 7.63 | 7.47 | 678 | | 50 | Yes | 30 |
| * No *G. pulex* was found at the site E1a, therefore the genetic and AOM toxicity analyses could not be performed for this site.  ** Site S1 is located downstream of the cascade of river dams, likely impassable for *G. pulex*. The distance from the source was therefore measured from the last dam before the first sampling site on the river.  *** Site W1 is located downstream of a large river dam, likely impassable for *G. pulex*. The distance from the source was therefore measured from this dam. | | | | | | | | | | |  |

Supplementary table S2. List of all detected compounds in *G. pulex* tissue and water samples with the application type of the compound, predicted logD values at pH 7.4, and, if available, EC_50_ value (EPA ecotoxicology database; https://www.epa.gov/chemical-research/ecotoxicology-database) used in the TU analysis.

See file: Tab_S2_list_analyzed_compounds.csv

Supplementary table S3. List of concentrations of compounds detected in water samples from every site in ng/L, with information on types of application (Type), and minimal detection limits (MDL) of the detected compounds.

See file: Tab_S3_list_water_compounds.csv

Supplementary table S4. List of concentrations of compounds detected in amphipod tissue samples from every site in ng/g wet tissue, with information on types of applications (Type), and minimal detection limits (MDL) of the detected compounds.

See file: Tab_S4_list_Gammarus_compounds.csv

Supplementary table S5. List of toxic units for compounds found in *G. pulex* tissue samples from every site.

See file: Tab_S5_list_Gammarus_TU.csv

Supplementary table S6. Primers used for the COI amplification, with information on primer name and reference, used concentration, and primer sequence.

| Primer name and reference | Concentration | Sequence |
| --- | --- | --- |
| LCO1490 (Folmer et al., 1994)  HC02198 (Folmer et al., 1994) | 1.0 M  1.0 M | GGTCAACAAATCATAAAGATATTGG  TAAACTTCAGGGTGACCAAAAAATCA |

Supplementary table S7. Primers used for microsatellite loci amplification including the information of primer code, forward (F) and reverse primer sequence, repeated sequence, M13 or CAG primer multiplex tag, multiplex reaction in which primer was used, and a publication reference.

| Code | Sequence | Repeat | Tag | Multi-plex | Ref. |
| --- | --- | --- | --- | --- | --- |
| gapu-8 | F:GAGCGTCATCATTTCCATCC  R:GCCAATCAGGGAACTGAGAA | (AT)^8 | No | 1 | Gergs et al., 2010 |
| Gapu-9 | F:CTATGCCCAAGCTGGTTGTT  R:TTCGCGTCATTCACTCGTAG | (ATT)^9 | No | 1 | Gergs et al., 2010 |
| Gapu-23 | F:CAGCAAGTGGTGCAGCTAAA  R:CAGCCACATCGAAGCTGTAA | (GCA)^11 | No | 1 | Gergs *et al.*. 2010 |
| Gapu-29 | F:CCTGCTCAGTAACAGCCTCA  R:TCAAATCGAGAAGGCTACAACA | (TTAA)^4/(AT)^4 | No | 2 | Gergs et al., 2010 |
| Gapu-30 | F:AAGTCGTTGCCATTGCTCTC  R:TCTTGGAGAGGGTGAGGTTG | (GT)^5/(ACA)5/(CAA)4+5 | No | 2 | Gergs et al., 2010 |
| Gammfos28 | F:ACCTCTCCATCCCTGATGC  R:CATCGACCCGTCAGTATGTG | (AC)13 | No | 2 | Westram et al., 2010 |
| Gp10 | F:TGAAATCGCACCCACTTCG  R:AGCTTCCAACAAGATTCCACC | (AC)^18 | M13 | 1 | Švara et al., 2019 |
| Gp11 | F:CATGCGCGACTAACCAGAC  R:GGATGACTGCCATGTGTACC | (ACT)^14 | M13 | 1 | Švara et al., 2019 |
| Gp13 | F:GGGAATTTGGCCTAGCGTATG  R:TGCAGTGGAGATGGTAGTCG | (TA)^22 | M13 | 1 | Švara et al., 2019 |
| Gp18 | F:GCACCATGGAGTCGATTTAGG  R:AAGTCATTGCTTGACGACGG | (ATT)^9 | M13 | 1 | Švara et al., 2019 |
| Gp28 | F:TTGTAGACCCGGCACATCC  R:TTCCCACGGATCTTGCACC | (AC)^12 | M13 | 2 | Švara et al., 2019 |
| Gp30 | F:AAACGACACAGTCTTGACTTC  R:CCCTTCTTTATACCAAATAACATTGCG | (AT)^22 | CAG | 1 | Švara et al., 2019 |
| Gp31 | F:CCTAACTAGGGGGAATCGGC  R:TGTCACACGAGACCCTGATG | (ATAC)^7 | M13 | 3 | Švara et al., 2019 |
| Gp37 | F:TGGGTATGTTTCGAATGATGTCTAC  R:TCCCTGCTCTAAGAAATTTGCG | (AT)^14 | M13 | 3 | Švara et al., 2019 |
| Gp42 | F:GTAAGCTCAACTCCACGGC  R:TCATGGTTGTAATGTTTGGATCAG | (AAT)^8 | CAG | 3 | Švara et al., 2019 |
| Gp55 | F:CCACATCTGGTCTACACTGGG  R:TGCGGACGCAAAGATGAAC | (AAC)^11 | M13 | 2 | Švara et al., 2019 |
| Gp68 | F:TAACCTTGGGTGAGTGGCAG  R:CCACCAGCGATTGTATGCAC | (ACGG)^8 | CAG | 2 | Švara et al., 2019 |

Supplementary table S8. List of parameters of genetic diversity analysis including sampling site name, number of genotyped amphipods (N), distance of sampling site from the river source (Distance (km)), rarefied allelic richness (AR), rarified private alleles (PA), average inbreeding coefficient of all individuals at each site (F_is_), observed heterozygosity (H_o_), expected heterozygosity (H_e_), and effective population size (N_e_).

| Site | N | Distance (km) | AR | PA | F_is_ | H_o_ | H_e_ | N_e_ |
| --- | --- | --- | --- | --- | --- | --- | --- | --- |
| A1 | 30 | 3.2 | 2.74 | 0.17 | 0.082 | 0.33 | 0.38 | 236.2 |
| A2 | 30 | 8.4 | 2.85 | 0.18 | 0.117 | 0.37 | 0.43 | 27.3 |
| A3 | 30 | 8.9 | 2.83 | 0.27 | 0.233 | 0.30 | 0.39 | 215.4 |
| A4 | 30 | 13.8 | 2.8 | 0.2 | 0.131 | 0.33 | 0.40 | 287.2 |
| A5 | 30 | 23.5 | 2.89 | 0.32 | 0.155 | 0.32 | 0.39 | 118 |
| E1 | 29 | 3.6 | 2.34 | 0.11 | 0.247 | 0.31 | 0.43 | 26.7 |
| E2 | 30 | 19 | 2.46 | 0.08 | 0.267 | 0.30 | 0.38 | 61.3 |
| E3 | 30 | 34.3 | 2.7 | 0.17 | 0.243 | 0.33 | 0.40 | 70.9 |
| E4 | 30 | 40.3 | 2.7 | 0.13 | 0.188 | 0.31 | 0.41 | 120.8 |
| E5 | 30 | 41.4 | 2.65 | 0.25 | 0.181 | 0.29 | 0.38 | 503.3 |
| E6 | 30 | 42.7 | 2.89 | 0.31 | 0.282 | 0.29 | 0.36 | 45 |
| H1 | 19 | 8 | 2.77 | 0.22 | -0.017 | 0.37 | 0.38 | 27.4 |
| H2 | 19 | 15 | 2.58 | 0.15 | 0.026 | 0.36 | 0.36 | 55.3 |
| H3 | 20 | 17 | 2.6 | 0.2 | 0.116 | 0.35 | 0.37 | 45.2 |
| H4 | 20 | 31 | 2.66 | 0.28 | 0.148 | 0.33 | 0.36 | 186.5 |
| H5 | 20 | 38 | 2.88 | 0.29 | 0.144 | 0.34 | 0.39 | 149.7 |
| H6 | 20 | 42 | 2.43 | 0.16 | 0.243 | 0.26 | 0.36 | 30.2 |
| P1 | 30 | 4.6 | 2.75 | 0.12 | 0.227 | 0.31 | 0.41 | 47.8 |
| P2 | 30 | 10.4 | 2.88 | 0.26 | 0.19 | 0.31 | 0.40 | 76.4 |
| P3 | 30 | 32.2 | 2.99 | 0.16 | 0.259 | 0.33 | 0.44 | 54.3 |
| P4 | 30 | 34.2 | 2.87 | 0.07 | 0.22 | 0.33 | 0.42 | 51.2 |
| P5 | 30 | 38.4 | 2.77 | 0.12 | 0.239 | 0.29 | 0.41 | 52.3 |
| S1 | 30 | 7 | 2.76 | 0.21 | 0.073 | 0.40 | 0.43 | 40.2 |
| S2 | 30 | 18.7 | 2.88 | 0.22 | 0.115 | 0.35 | 0.42 | 22.3 |
| S3 | 30 | 25.3 | 2.81 | 0.2 | 0.182 | 0.36 | 0.43 | 39.4 |
| S4 | 30 | 27.4 | 2.79 | 0.23 | 0.174 | 0.35 | 0.42 | 108.9 |
| S5 | 30 | 29.1 | 2.94 | 0.26 | 0.17 | 0.36 | 0.44 | 371.2 |
| W1 | 30 | 4.5 | 2.64 | 0.18 | 0.269 | 0.30 | 0.40 | 118.9 |
| W2 | 30 | 8.8 | 2.77 | 0.14 | 0.121 | 0.31 | 0.40 | 113.8 |
| W3 | 30 | 19.3 | 2.85 | 0.17 | 0.161 | 0.31 | 0.40 | 1371.3 |
| W4 | 30 | 24.8 | 2.79 | 0.21 | 0.439 | 0.27 | 0.39 | 71 |
| W5 | 21 | 29.3 | 2.78 | 0.23 | ß.245 | 0.29 | 0.38 | 20.8 |
| W6 | 10 | 33.1 | 2.61 | 0.12 | 0.226 | 0.23 | 0.35 | 14.3 |
| W7 | 30 | 34.9 | 2.84 | 0.19 | 0.2 | 0.31 | 0.39 | 34 |

Supplementary table S9. Null allele rates (Null) and total detected alleles (Alleles) across sampled loci and rivers.

|  | Altenau | | Eine | | Holtemme | | Parthe | | Saale | | Wipper | |
| --- | --- | --- | --- | --- | --- | --- | --- | --- | --- | --- | --- | --- |
| Locus | Null | Alleles | Null | Alleles | Null | Alleles | Null | Alleles | Null | Alleles | Null | Alleles |
| g8 | 0.09 | 4 | 0.34 | 5 | 0.06 | 5 | 0.05 | 4 | 0.15 | 6 | 0.12 | 4 |
| g9 | 0 | 4 | 0 | 1 | 0 | 2 | 0 | 1 | 0 | 2 | 0.05 | 6 |
| g23 | 0 | 5 | 0 | 6 | 0 | 4 | 0.1 | 4 | 0 | 4 | 0 | 6 |
| g29 | 0 | 2 | 0 | 4 | 0 | 3 | 0.06 | 2 | 0.19 | 3 | 0 | 2 |
| g30 | 0 | 1 | 0 | 1 | 0 | 5 | 0 | 1 | 0 | 1 | 0 | 3 |
| gf28 | 0 | 4 | 0.11 | 2 | 0 | 2 | 0 | 1 | 0 | 4 | 0 | 3 |
| gp10 | 0 | 10 | 0 | 11 | 0 | 9 | 0.06 | 9 | 0 | 12 | 0.15 | 11 |
| gp11 | 0 | 8 | 0.05 | 6 | 0.07 | 5 | 0.06 | 6 | 0 | 3 | 0.05 | 5 |
| gp18 | 0.1 | 3 | 0 | 4 | 0 | 4 | 0 | 5 | 0 | 3 | 0.08 | 4 |
| gp28 | 0 | 12 | 0 | 11 | 0 | 11 | 0.05 | 10 | 0 | 12 | 0 | 13 |
| gp30 | 0.14 | 6 | 0.13 | 4 | 0 | 4 | 0.15 | 5 | 0.18 | 5 | 0.18 | 5 |
| gp31 | 0 | 4 | 0 | 4 | 0 | 5 | 0 | 5 | 0 | 5 | 0 | 5 |
| gp37 | 0.28 | 12 | 0.47 | 9 | 0.36 | 9 | 0.51 | 9 | 0.43 | 9 | 0.33 | 10 |
| gp42 | 0.8 | 3 | 0 | 2 | 0 | 3 | 0.05 | 2 | 0.07 | 3 | 0 | 4 |
| gp55 | 0 | 5 | 0.08 | 5 | 0 | 5 | 0.19 | 4 | 0 | 8 | 0.13 | 7 |
| gp68 | 0 | 9 | 0 | 9 | 0 | 7 | 0 | 7 | 0 | 7 | 0 | 7 |

Supplementary table S10. Tables of F_st_ values from pairwise comparison between sites within rivers. The significant values are listed on a grey background, while the non-significant values are listed on a red background.

|  | A1 | A2 | A3 | A4 | A5 | E1 | E2 | E3 | E4 | E5 | E6 | H1 | H2 | H3 | H4 | H5 | H6 | P1 | P2 | P3 | P4 | P5 | S1 | S2 | S3 | S4 | S5 | W1 | W2 | W3 | W4 | W5 | W6 | W7 |
| --- | --- | --- | --- | --- | --- | --- | --- | --- | --- | --- | --- | --- | --- | --- | --- | --- | --- | --- | --- | --- | --- | --- | --- | --- | --- | --- | --- | --- | --- | --- | --- | --- | --- | --- |
| A1 | 0 |  |  |  |  |  |  |  |  |  |  |  |  |  |  |  |  |  |  |  |  |  |  |  |  |  |  |  |  |  |  |  |  |  |
| A2 | 0.01 | 0 |  |  |  |  |  |  |  |  |  |  |  |  |  |  |  |  |  |  |  |  |  |  |  |  |  |  |  |  |  |  |  |  |
| A3 | 0.02 | 0.01 | 0 |  |  |  |  |  |  |  |  |  |  |  |  |  |  |  |  |  |  |  |  |  |  |  |  |  |  |  |  |  |  |  |
| A4 | 0.02 | 0.01 | 0.01 | 0 |  |  |  |  |  |  |  |  |  |  |  |  |  |  |  |  |  |  |  |  |  |  |  |  |  |  |  |  |  |  |
| A5 | 0.03 | 0.02 | 0.01 | 0.01 | 0 |  |  |  |  |  |  |  |  |  |  |  |  |  |  |  |  |  |  |  |  |  |  |  |  |  |  |  |  |  |
| E1 | 0.21 | 0.2 | 0.2 | 0.18 | 0.19 | 0 |  |  |  |  |  |  |  |  |  |  |  |  |  |  |  |  |  |  |  |  |  |  |  |  |  |  |  |  |
| E2 | 0.2 | 0.19 | 0.18 | 0.17 | 0.18 | 0.04 | 0 |  |  |  |  |  |  |  |  |  |  |  |  |  |  |  |  |  |  |  |  |  |  |  |  |  |  |  |
| E3 | 0.18 | 0.17 | 0.17 | 0.15 | 0.16 | 0.05 | 0.01 | 0 |  |  |  |  |  |  |  |  |  |  |  |  |  |  |  |  |  |  |  |  |  |  |  |  |  |  |
| E4 | 0.19 | 0.18 | 0.17 | 0.16 | 0.18 | 0.04 | 0 | 0.01 | 0 |  |  |  |  |  |  |  |  |  |  |  |  |  |  |  |  |  |  |  |  |  |  |  |  |  |
| E5 | 0.2 | 0.19 | 0.18 | 0.17 | 0.17 | 0.05 | 0 | 0 | 0 | 0 |  |  |  |  |  |  |  |  |  |  |  |  |  |  |  |  |  |  |  |  |  |  |  |  |
| E6 | 0.15 | 0.14 | 0.13 | 0.12 | 0.13 | 0.08 | 0.02 | 0.01 | 0 | 0.01 | 0 |  |  |  |  |  |  |  |  |  |  |  |  |  |  |  |  |  |  |  |  |  |  |  |
| H1 | 0.16 | 0.16 | 0.17 | 0.14 | 0.14 | 0.09 | 0.1 | 0.09 | 0.09 | 0.08 | 0.1 | 0 |  |  |  |  |  |  |  |  |  |  |  |  |  |  |  |  |  |  |  |  |  |  |
| H2 | 0.17 | 0.17 | 0.16 | 0.15 | 0.15 | 0.07 | 0.06 | 0.07 | 0.06 | 0.06 | 0.07 | 0.02 | 0 |  |  |  |  |  |  |  |  |  |  |  |  |  |  |  |  |  |  |  |  |  |
| H3 | 0.15 | 0.15 | 0.14 | 0.13 | 0.13 | 0.05 | 0.06 | 0.06 | 0.06 | 0.05 | 0.06 | 0 | 0 | 0 |  |  |  |  |  |  |  |  |  |  |  |  |  |  |  |  |  |  |  |  |
| H4 | 0.18 | 0.18 | 0.17 | 0.16 | 0.16 | 0.07 | 0.07 | 0.08 | 0.07 | 0.06 | 0.08 | 0.03 | 0 | 0 | 0 |  |  |  |  |  |  |  |  |  |  |  |  |  |  |  |  |  |  |  |
| H5 | 0.14 | 0.14 | 0.13 | 0.11 | 0.11 | 0.08 | 0.08 | 0.08 | 0.07 | 0.07 | 0.07 | 0.01 | 0 | 0 | 0 | 0 |  |  |  |  |  |  |  |  |  |  |  |  |  |  |  |  |  |  |
| H6 | 0.11 | 0.12 | 0.1 | 0.09 | 0.08 | 0.09 | 0.1 | 0.08 | 0.09 | 0.08 | 0.08 | 0.03 | 0.03 | 0.02 | 0.05 | 0.02 | 0 |  |  |  |  |  |  |  |  |  |  |  |  |  |  |  |  |  |
| P1 | 0.12 | 0.1 | 0.09 | 0.11 | 0.11 | 0.17 | 0.18 | 0.15 | 0.16 | 0.15 | 0.12 | 0.15 | 0.14 | 0.12 | 0.14 | 0.12 | 0.13 | 0 |  |  |  |  |  |  |  |  |  |  |  |  |  |  |  |  |
| P2 | 0.14 | 0.12 | 0.11 | 0.13 | 0.12 | 0.16 | 0.17 | 0.14 | 0.14 | 0.14 | 0.11 | 0.12 | 0.11 | 0.09 | 0.11 | 0.1 | 0.1 | 0.03 | 0 |  |  |  |  |  |  |  |  |  |  |  |  |  |  |  |
| P3 | 0.12 | 0.11 | 0.1 | 0.12 | 0.1 | 0.13 | 0.14 | 0.1 | 0.12 | 0.11 | 0.08 | 0.1 | 0.1 | 0.07 | 0.09 | 0.08 | 0.08 | 0.03 | 0.02 | 0 |  |  |  |  |  |  |  |  |  |  |  |  |  |  |
| P4 | 0.1 | 0.09 | 0.07 | 0.1 | 0.08 | 0.14 | 0.15 | 0.11 | 0.14 | 0.13 | 0.1 | 0.11 | 0.11 | 0.08 | 0.11 | 0.09 | 0.07 | 0.04 | 0.02 | 0 | 0 |  |  |  |  |  |  |  |  |  |  |  |  |  |
| P5 | 0.12 | 0.1 | 0.09 | 0.11 | 0.09 | 0.16 | 0.17 | 0.13 | 0.15 | 0.14 | 0.12 | 0.12 | 0.13 | 0.1 | 0.13 | 0.1 | 0.08 | 0.05 | 0.03 | 0 | 0 | 0 |  |  |  |  |  |  |  |  |  |  |  |  |
| S1 | 0.22 | 0.18 | 0.18 | 0.17 | 0.16 | 0.21 | 0.2 | 0.17 | 0.18 | 0.2 | 0.14 | 0.2 | 0.21 | 0.18 | 0.21 | 0.18 | 0.19 | 0.17 | 0.16 | 0.14 | 0.14 | 0.16 | 0 |  |  |  |  |  |  |  |  |  |  |  |
| S2 | 0.16 | 0.13 | 0.12 | 0.12 | 0.1 | 0.23 | 0.22 | 0.17 | 0.2 | 0.2 | 0.14 | 0.19 | 0.2 | 0.17 | 0.21 | 0.17 | 0.16 | 0.15 | 0.13 | 0.11 | 0.1 | 0.12 | 0.04 | 0 |  |  |  |  |  |  |  |  |  |  |
| S3 | 0.18 | 0.15 | 0.14 | 0.14 | 0.13 | 0.24 | 0.22 | 0.18 | 0.2 | 0.21 | 0.15 | 0.21 | 0.22 | 0.19 | 0.23 | 0.19 | 0.18 | 0.16 | 0.15 | 0.13 | 0.12 | 0.15 | 0.02 | 0 | 0 |  |  |  |  |  |  |  |  |  |
| S4 | 0.16 | 0.15 | 0.14 | 0.12 | 0.12 | 0.22 | 0.21 | 0.16 | 0.19 | 0.19 | 0.13 | 0.19 | 0.21 | 0.18 | 0.21 | 0.17 | 0.16 | 0.17 | 0.16 | 0.14 | 0.14 | 0.16 | 0.08 | 0.02 | 0.03 | 0 |  |  |  |  |  |  |  |  |
| S5 | 0.15 | 0.11 | 0.12 | 0.1 | 0.1 | 0.21 | 0.2 | 0.16 | 0.18 | 0.18 | 0.13 | 0.17 | 0.2 | 0.17 | 0.21 | 0.16 | 0.15 | 0.15 | 0.13 | 0.12 | 0.11 | 0.13 | 0.06 | 0.01 | 0.02 | 0.01 | 0 |  |  |  |  |  |  |  |
| W1 | 0.11 | 0.08 | 0.05 | 0.09 | 0.09 | 0.2 | 0.15 | 0.14 | 0.13 | 0.16 | 0.09 | 0.19 | 0.15 | 0.15 | 0.17 | 0.14 | 0.14 | 0.11 | 0.1 | 0.11 | 0.07 | 0.1 | 0.15 | 0.11 | 0.11 | 0.13 | 0.1 | 0 |  |  |  |  |  |  |
| W2 | 0.09 | 0.08 | 0.05 | 0.07 | 0.08 | 0.15 | 0.13 | 0.1 | 0.11 | 0.12 | 0.07 | 0.14 | 0.12 | 0.1 | 0.13 | 0.11 | 0.09 | 0.08 | 0.06 | 0.07 | 0.04 | 0.07 | 0.15 | 0.1 | 0.11 | 0.11 | 0.09 | 0.02 | 0 |  |  |  |  |  |
| W3 | 0.13 | 0.11 | 0.08 | 0.1 | 0.09 | 0.19 | 0.12 | 0.11 | 0.1 | 0.11 | 0.06 | 0.17 | 0.14 | 0.13 | 0.15 | 0.12 | 0.12 | 0.12 | 0.1 | 0.11 | 0.08 | 0.1 | 0.15 | 0.1 | 0.11 | 0.11 | 0.09 | 0.02 | 0.03 | 0 |  |  |  |  |
| W4 | 0.14 | 0.11 | 0.09 | 0.11 | 0.09 | 0.24 | 0.18 | 0.15 | 0.16 | 0.16 | 0.11 | 0.21 | 0.19 | 0.17 | 0.2 | 0.16 | 0.15 | 0.14 | 0.13 | 0.13 | 0.11 | 0.13 | 0.16 | 0.09 | 0.11 | 0.11 | 0.09 | 0.04 | 0.05 | 0 | 0 |  |  |  |
| W5 | 0.13 | 0.1 | 0.08 | 0.12 | 0.1 | 0.25 | 0.17 | 0.15 | 0.16 | 0.16 | 0.1 | 0.22 | 0.19 | 0.18 | 0.2 | 0.18 | 0.16 | 0.14 | 0.11 | 0.12 | 0.1 | 0.11 | 0.19 | 0.12 | 0.14 | 0.15 | 0.12 | 0.03 | 0.05 | 0.01 | 0 | 0 |  |  |
| W6 | 0.13 | 0.11 | 0.09 | 0.12 | 0.12 | 0.27 | 0.2 | 0.17 | 0.19 | 0.19 | 0.12 | 0.25 | 0.22 | 0.2 | 0.22 | 0.19 | 0.18 | 0.15 | 0.14 | 0.14 | 0.12 | 0.14 | 0.21 | 0.13 | 0.16 | 0.16 | 0.14 | 0.06 | 0.05 | 0.04 | 0.03 | 0.01 | 0 |  |
| W7 | 0.12 | 0.1 | 0.08 | 0.09 | 0.1 | 0.24 | 0.18 | 0.16 | 0.16 | 0.17 | 0.11 | 0.2 | 0.17 | 0.16 | 0.18 | 0.15 | 0.14 | 0.13 | 0.12 | 0.14 | 0.11 | 0.14 | 0.19 | 0.12 | 0.14 | 0.13 | 0.12 | 0.04 | 0.04 | 0.02 | 0.02 | 0.02 | 0 | 0 |

Supplementary table S11. Linear mixed-effect models for analyzed parameters with selected fixed effects (Distance from source (Dist), WWTP effluent upstream of the sampling site (WWTP), total concentration of AOM (Total), toxic unit values (TU), oxygen concentration (Oxy), pH value (pH), and conductivity (Cond)) and selection criteria (log Likelihood (logLik) and AIC criterion (AICc)).

| Indicator | Predictors (Fixed effects) | | | | | | | df | logLik | AICc |
| --- | --- | --- | --- | --- | --- | --- | --- | --- | --- | --- |
|  | Dist | WWTP | Total | TU | Oxy | pH | Cond |  |  |  |
| Allelic Richness |  |  |  |  |  |  |  | 3 | 14.609 | -22.3 |
|  |  | + | + |  |  |  |  | 5 | 17.255 | -22.1 |
|  |  | + |  |  |  |  |  | 4 | 14.512 | -19.5 |
|  |  |  | + |  |  |  |  | 4 | 14.160 | -18.8 |
|  |  |  |  | + |  |  |  | 4 | 13.902 | -18.3 |
| Inbreeding (F_is_) |  |  |  |  |  |  |  | 3 | 31.022 | -55.2 |
|  |  |  |  | + |  |  |  | 4 | 30.814 | -52.1 |
|  |  | + |  |  |  |  |  | 4 | 30.716 | -51.9 |
| Private alleles |  |  |  |  |  |  |  | 3 | 37.929 | -69.0 |
| Abundance |  | + | + |  |  |  |  | 5 | -17.400 | 47.2 |
|  |  |  | + | + |  |  |  | 5 | -17.834 | 48.1 |
|  |  |  | + |  |  |  |  | 4 | -19.995 | 49.5 |
|  | + |  | + |  |  |  |  | 5 | -18.610 | 49.6 |
|  |  | + | + | + |  |  |  | 6 | -17.375 | 50.3 |
|  |  |  |  |  |  |  |  | 3 | -22.337 | 51.6 |
|  |  | + | + |  |  | + |  | 6 | -18.168 | 51.8 |
| N_~~e~~_ |  |  |  |  |  |  |  | 3 | -17.708 | 42.3 |
|  |  | + |  |  |  |  |  | 4 | -17.031 | 43.6 |
|  |  | + |  | + |  |  |  | 5 | -16.909 | 46.2 |
|  |  |  | + |  |  |  |  | 4 | -18.437 | 46.4 |
|  |  |  |  |  |  | + |  | 4 | -18.456 | 46.5 |
|  |  |  |  | + |  |  |  | 4 | -18.735 | 47.0 |

Supplementary tables S12. Structural equation model output for each river and all rivers combined. A list of the analyzed parameters with a model estimate, standard errors (SE), z-values (z), and p-values (p) are included.

| Wipper | Estimate | SE | z | p |
| --- | --- | --- | --- | --- |
| Allel_Rich | ~ |  |  |  |
| Distance_sourc | 0.006 | 0.002 | 2.579 | 0.010 |
| Total_log | -0.055 | 0.118 | -0.470 | 0.639 |
| Total_log | ~ |  |  |  |
| Distance_sourc | 0.010 | 0.007 | 1.366 | 0.172 |

| Saale | Estimate | SE | z | p |
| --- | --- | --- | --- | --- |
| Allel_Rich | ~ |  |  |  |
| Distance_sourc | 0.010 | 0.002 | 4.297 | 0.000 |
| Total_log | -0.389 | 0.127 | -3.062 | 0.002 |
| Total_log | ~ |  |  |  |
| Distance_sourc | 0.014 | 0.006 | 2.114 | 0.034 |

| Parthe | Estimate | SE | z | p |
| --- | --- | --- | --- | --- |
| Allel_Rich | ~ |  |  |  |
| Distance_sourc | 0.004 | 0.005 | 0.791 | 0.429 |
| Total_log | -0.164 | 0.341 | -0.483 | 0.629 |
| Total_log | ~ |  |  |  |
| Distance_sourc | 0.012 | 0.003 | 3.337 | 0.001 |

| Holtemme | Estimate | SE | z | p |
| --- | --- | --- | --- | --- |
| Allel_Rich | ~ |  |  |  |
| Distance_sourc | 0.001 | 0.006 | 0.144 | 0.886 |
| Total_log | -0.068 | 0.116 | -0.584 | 0.559 |
| Total_log | ~ |  |  |  |
| Distance_sourc | 0.039 | 0.016 | 2.474 | 0.013 |

| Eine | Estimate | SE | z | p |
| --- | --- | --- | --- | --- |
| Allel_Rich | ~ |  |  |  |
| Distance_sourc | 0.010 | 0.001 | 8.191 | 0.000 |
| Total_log | -0.428 | 0.121 | -3.531 | 0.000 |
| Total_log | ~ |  |  |  |
| Distance_sourc | -0.003 | 0.004 | -0.894 | 0.371 |

| Altenau | Estimate | SE | z | p |
| --- | --- | --- | --- | --- |
| Allel_Rich | ~ |  |  |  |
| Distance_sourc | 0.006 | 0.002 | 2.896 | 0.004 |
| Total_log | -0.085 | 0.097 | -0.879 | 0.380 |
| Total_log | ~ |  |  |  |
| Distance_sourc | -0.001 | 0.009 | -0.140 | 0.889 |
|  |  |  |  |  |
| All | **Estimate** | **SE** | **z** | **p** |
| Allel_Rich | ~ |  |  |  |
| Distance_sourc | 0.005 | 0.002 | 2.357 | 0.018 |
| Abund | 0.125 | 0.051 | 2.434 | 0.015 |
| TUgam | 0.033 | 0.015 | 2.222 | 0.026 |
| Total_log | -0.257 | 0.056 | -4.588 | 0.000 |
| Abund | ~ |  |  |  |
| Distance_sourc | -0.022 | 0.006 | -3.498 | 0.000 |
| TUgam | 0.008 | 0.052 | 0.156 | 0.876 |
| Total_log | 0.630 | 0.161 | 3.924 | 0.000 |
| TUgam | ~ |  |  |  |
| Distance_sourc | 0.057 | 0.019 | 2.981 | 0.003 |
| Total_log | -0.182 | 0.556 | -0.328 | 0.743 |
| Total_log | ~ |  |  |  |
| Distance_sourc | 0.013 | 0.006 | 2.372 | 0.018 |

**Literature in Supporting Information**

Applied Biosystems. (2010). Purifying extension products. In BigDye terminator v3.1 Cycle sequencing kit. USA.

Beckers, L. M., Brack, W., Dann, J. P., Krauss, M., Müller, E., & Schulze, T. (2020). Unraveling longitudinal pollution patterns of organic micropollutants in a river by non-target screening and cluster analysis. *Science of the Total Environment, 727*, 138388. https://doi.org/10.1016/j.scitotenv.2020.138388

Folmer, O., Black, M., Hoeh, W., Lutz, R., & Vrijenhoek, R. (1994). DNA primers for amplification of mitochondrial cytochrome c oxidase subunit I from diverse metazoan invertebrates*. Molecular Marine Biology and Biotechnology, 3(5)*, 294–299.

Gergs, R., Rothhaupt, K.-O., & Behrmann-Godel, J. (2010). Characterisation of polymorphic microsatellite markers for the freshwater amphipod *Gammarus pulex* L. (Crustacea: Amphipoda). *Molecular Ecology Resources, 10(1)*, 232–236. https://doi.org/10.1111/j.1755-0998.2009.02796.x

Inostroza, P. A., Wicht, A. J., Huber, T., Nagy, C., Brack, W., & Krauss, M. (2016). Body burden of pesticides and wastewater-derived pollutants on freshwater invertebrates: Method development and application in the Danube River. *Environmental Pollution, 214*, 77–85. https://doi.org/10.1016/j.envpol.2016.03.064

Kumar, S., Nei, M., Dudley, J., & Tamura, K. (2008). MEGA: A biologist-centric software for evolutionary analysis of DNA and protein sequences. *Briefings in Bioinformatics, 9(4)*, 299–306. https://doi.org/10.1093/bib/bbn017

Pluskal, T., Castillo, S., Villar-Briones, A., & Orešič, M. (2010). MZmine 2: Modular framework for processing, visualizing, and analyzing mass spectrometry-based molecular profile data. *BMC Bioinformatics, 11*. https://doi.org/10.1186/1471-2105-11-395

Švara, V., Norf, H., Luckenbach, T., Brack, W., & Michalski, S. G. (2019). Isolation and characterization of eleven novel microsatellite markers for fine‑scale population genetic analyses of *Gammarus pulex* (Crustacea: Amphipoda). *Molecular Biology Reports, 46(6)*, 6609–6615. https://doi.org/10.1007/s11033-019-05077-y

Tamura, K., Peterson, D., Peterson, N., Stecher, G., Nei, M., & Kumar, S. (2011). MEGA5: Molecular evolutionary genetics analysis using maximum likelihood, evolutionary distance, and maximum parsimony methods. *Molecular Biology and Evolution, 28(10)*, 2731–2739. https://doi.org/10.1093/molbev/msr121

Westram, A. M., Jokela, J., & Keller, I. (2010). Isolation and characterization of ten polymorphic microsatellite markers for three cryptic *Gammarus fossarum* (Amphipoda) species. *Conservation Genetics Resources, 2(1)*, 401–404. https://doi.org/10.1007/s12686-010-9287-1
